# Research and Clinical Landscape of Bispecific Antibodies for the Treatment of Solid Malignancies

**DOI:** 10.3390/ph14090884

**Published:** 2021-08-31

**Authors:** Gabriele Antonarelli, Federica Giugliano, Chiara Corti, Matteo Repetto, Paolo Tarantino, Giuseppe Curigliano

**Affiliations:** 1 Division of Early Drug Development for Innovative Therapy, European Institute of Oncology, IRCCS, 20141 Milan, Italy; gabriele.antonarelli@ieo.it (G.A.); federica.giugliano@ieo.it (F.G.); chiara.corti@ieo.it (C.C.); matteo.repetto@ieo.it (M.R.); paolo.tarantino@ieo.it (P.T.); 2 Department of Oncology and Haematology (DIPO), University of Milan, 20122 Milan, Italy

**Keywords:** bispecific, antibodies, solid, tumors, immunotherapy

## Abstract

Solid tumors adopt multiple mechanisms to grow, evade immune responses, and to withstand therapeutic approaches. A major breakthrough in the armamentarium of anti-cancer agents has been the introduction of monoclonal antibodies (mAbs), able to inhibit aberrantly activated pathways and/or to unleash antigen (Ag)-specific immune responses. Nonetheless, mAb-mediated targeted pressure often fails due to escape mechanisms, mainly Ag loss/downregulation, ultimately providing therapy resistance. Hence, in order to target multiple Ag at the same time, and to facilitate cancer-immune cells interactions, bispecific antibodies (bsAbs) have been developed and are being tested in clinical trials, yielding variable safety/efficacy results based on target selection and their structure. While in hematologic cancers the bsAb blinatumomab recently reached the Food and Drug Administration (FDA)-approval for B Cell Acute Lymphoblastic Leukemia, bsAbs use in solid tumors faces considerable challenges, such as target Ag selection, biodistribution, and the presence of an immune-suppressive tumor microenvironment (TME). This review will focus on the state-of-the art, the design, and the exploitation of bsAbs against solid malignancies, delineating their mechanisms of action, major pitfalls, and future directions.

## 1. Introduction

Immunotherapy has recently changed both the treatment modalities as well as the prognosis across several malignancies. Such immune-stimulating therapies entail either passive (i.e., monoclonal antibodies, adoptive cell therapies, cytokines) or active (i.e., cancer vaccines, oncolytic viruses, checkpoint blockade) approaches [1,2]. Clinical responses to immunotherapies are vastly heterogeneous, according to patient-, tumor-, or treatment-related characteristics. In this context, the ability of eliciting Ag-specific anti-cancer immune responses has been linked with impressive and durable responses in several malignancies. For example, chimeric antigen receptor (CAR) T cells therapies have been approved in different hematologic tumors, inducing complete/partial remissions in high-tumor burden patients [3,4]. However, CAR-T cells manufacture, costs, and scalability are some of the factors that partially limit their widespread use.

Monoclonal antibodies (mAb) represent another way to induce Ag-specific immune responses. Differently from CAR-T cell therapies, mAbs have the advantage of being ready, off-the-shelf, drug products with a potentially safer therapeutic index, also considering chemotherapy conditioning for CAR-T cell therapies. Clinical results with mAb, especially against hematologic malignancies, further sped up development of the entire field [5,6]. Nowadays, key areas of research are represented by antigen discovery (both on cancer cells and on immune/stromal cells), antibody design (i.e., to improve pharmacodynamics and pharmacokinetics, or to allow for drug-conjugation) and combinatorial strategies. In this scenario, with the aim to tackle evasion mechanisms adopted by tumors upon strong therapeutic pressure, the production of bispecific antibodies (bsAbs) became a reality thanks to protein engineering advancements. Today, more than 100 bsAb formats have been developed and a growing number of them have entered clinical testing over recent years [7]. In this review, we will summarize major characteristics of bsAbs underlying their activities, we will discuss the most advanced drug products tested against solid malignancies and provide an overview on the potential future advancements in the bsAb field.

## 2. Bispecific Antibodies Design and Development

The advent of mAbs for cancer treatment has drastically changed clinical practice, leading to improved survival outcomes in several malignancies [8]. They are made of two heterodimers composed by two identical heavy and light chains, with two Fragment Ab-binding (Fab; one variable and one constant domain of the heavy and light chains) and one Fragment Crystallizable region (Fc, made of the second and third constant domains of the heavy chains). In 1975, the landmark development of the “hybridoma” technology by Köhler and Milstein led to the production of large quantities of mAbs, fusing B cells from an Ag-exposed mice with immortalized myeloma cells [9]. Over the years, mAbs have evolved to improve their design and reduce undesired immunogenicity of their structure primarily by humanization processes [10].

Subsequently, bsAbs soon became an attractive area for drug development purposes. A key challenge is represented by incorrect protein heterodimerization in the production pipeline: this may involve both heavy and light chains mispairing, which may account for up to 90% of the total mass [11]. To overcome this issue, purification methodologies involving either affinity, size, hydrophobicity, charge, or mixed-based methods are employed during bsAb production [12]. The first attempt to produce heterodimeric bsAbs involved the fusion of two different hybridoma cell lines, the “hybrid hybridoma” (quadroma) technology. Despite the pioneering implications, quadromas are characterized by intrinsic Ig chain mispairing with a 1:10 ratio between the desired/produced bsAbs [13]. In 1997, the introduction of the Knob-in-Hole (KIH) technology solved the issue of heavy chain mispairing by introducing mutations in the constant heavy 3 (CH3) domains, improving the desired/produced bsAbs ratio to 1:4 [14]. Since then, numerous novel technologies have emerged, dictating diverse pharmacokinetic and pharmacodynamic profiles [15]. In parallel, the discovery of novel targets, either on transformed or healthy (i.e., immune/stromal cells) tissues, as well as novel payloads (i.e., cytokines or chemotherapeutics), have supported the increasing use and testing of bsAbs in the last decades [16].

BsAbs are categorized based on their structure and mechanisms of action (MoA), which are dictated by their specificity (number of recognized antigens, Ag), valency (total number of Ag-binding sites per molecule), and affinity (interaction strength). For instance, it has been shown that reducing CD3 affinity allows for better biodistribution in tumor sites (versus lymph nodes) and lower rates of cytokine release, while preserving T-cell engaging/activation properties [8,9,17,18]. Moreover, bsAbs may also exert their functions by increased steric hindrance properties compared to a mixture of two corresponding monospecific mAbs. Indeed, while such phenomenon may reduce bsAb-epitope binding properties, it has been shown to negatively impact receptor heterodimerization for ErbB2 and VEGFR-2–VEGF, ultimately resulting in improved tumor cell killing in pre-clinical models [19,20].

### 2.1. Structural Classification of Bispecific Antibodies

A key structural feature of bsAbs is represented by the presence/absence of the Fc region.

#### 2.1.1. Fc-Free Bispecific Antibodies

Fc-free bsAbs display better biodistribution into tumor tissues, higher potency, and less common incidence of immune-related adverse effects (irAEs) [21,22]. However, to achieve favorable PK profiles, Fc-free molecules need either continuous intravenous infusion or structural modifications to prolong their half-lives, such as fusion with polyethylene glycol or human serum albumin [23]. Available Fc-free bsAbs are mainly represented by bispecific T-cell engagers (BiTEs), dual affinity re-targeting (DARTs), and single-chain from the heavy variable domain (ScFv). One notable example is the anti-CD19/CD3 BiTE Blinatumomab, which was granted approval by the Food and Drug Administration (FDA) for relapsed/refractory B-cell precursor acute lymphoblastic leukemia (B-ALL) and for minimal residual disease (MRD) positive B-ALL patients in first/second complete remission [24]. Many Fc-free BiTEs are currently under clinical evaluation in solid tumors. For example, catumaxomab, an anti-CD3/EpCAM, was granted European Medical Agency (EMA) approval in 2009 for the intraperitoneal treatment of malignant ascites, but was later withdrawn from the market in 2013 for commercial reasons [25]. Nonetheless, catumaxomab is currently being investigated for diverse indications in large phase II–III studies (Table S1).

#### 2.1.2. Fc-Bearing Bispecific Antibodies

Fc-containing bsAbs, instead, are characterized by longer half-lives [23] and by their ability of eliciting effector functions on immune cells, such as antibody-dependent cell cytotoxicity (ADCC) or complement-dependent cytotoxicity (CDC) [26]. Fc-based bsAbs display either Immunoglobulin G (IgG)-like or, more commonly, IgG-modified structures [22].

IgG-like Fc-based bsAbs are characteristically limited by design to a fixed Ag specificity and a valency of two. IgG-like bsAbs comprise CrossMab 1:1, Triomabs and Fab-arm exchange (FAE) platforms. CrossMabs (i.e., vanucizumab) have two asymmetric Fab arms, because the desired light chain pairing is forced by swapping either the constant or the variable domains between heavy and light chains, creating two asymmetric Fab arms [27]. Triomabs (i.e., catumaxomab) exploit species-restricted heavy/light chain pairing [28], whereas FAEs (i.e., amivantamab) work by introducing complementary point mutations in the CH3 domain to force desired heterodimerization [29].

IgG-modified bsAbs, instead, have a fixed specificity of two but can have a valency of two or more. Examples of IgG-modified bsAbs are CrossMab 2:1 or 2:2 (i.e., cibisatamab), Half-Life Extended BiTEs (HLE-BiTEs) (i.e., AMG757), Dual Affinity Re-targeting (DART)-Fc (i.e., tebotelimab), Dual variable domain (DVD)-IgG (i.e., dilpacimab), and Fabs-in-tandem (FIT)-Ig (i.e., EMB-01). Of note, FIT-Ig are composed by two fused Fabs in a crisscross orientation, resulting in a tetravalent IgG-like molecule [30]. Instead, DVD-IgG specificity is guaranteed regardless of light chain mispairing since each chain contains two variable domains one after the other [31].

### 2.2. Mechanistic Classification of Bispecific Antibodies

**Figure 1:**
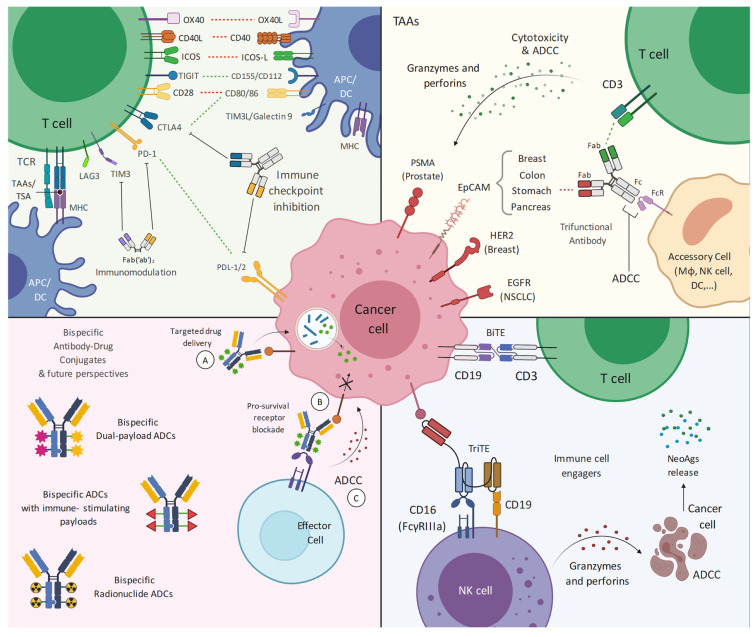
Main design, applications, and future perspectives of bispecific antibodies and engagers. Each quadrant represents different applications of bi- and tri-specific novel technologies. **Upper left panel**: dual immune checkpoint blockade by means of bispecific antibodies or nanobodies. Co-targeting of multiple co-stimulatory molecules has been exploited either to enhance immune checkpoint inhibition or to combine immune checkpoint blockade and immunomodulation. Red-dashed lines: co-stimulatory molecules; green-dashed lines: co-inhibitory molecules. **Upper right panel**: bispecific antibodies targeting dual tumor-associated antigens. Trifunctional properties of bsAbs comprise an appropriate Fc region in order to not only recruit T-cells, but also accessory cells bearing activating FcγR. Hence, additional T-cell-activating signals and presentation of tumor-derived antigens to T-cells can be provided; **Lower left panel**: bispecific antibody-drug conjugates are investigational strategies aiming at exploiting both the target-specific properties of engineered antibodies and the cytotoxic activity of payloads; **Lower right panel**: most immune cell engagers are trans-binding bispecific antibodies (bsAbs) usually consisting of two linked single-chain fragment variables (scFvs) that originate from different monoclonal antibodies: one scFv recognizes a surface TAA, whereas the other is specific for a certain membrane molecule expressed on effector immune cells. Abbreviations: OX40, Tumor necrosis factor receptor superfamily, member 4 or TNFRSF4; OX40L, OX40 ligand; ICOS, Inducible costimulator; ICOS-L, Inducible costimulator-ligand (ICOS-L); CD, cluster of differentiation; TIGIT, T cell immunoreceptor with Ig and ITIM domains; TIM3, T-cell immunoglobulin mucin 3; TIM3L, T-cell immunoglobulin mucin 3 ligand; CTLA-4, Cytotoxic T-Lymphocyte Antigen 4; PD-L1, Programmed death-ligand 1; PD-1, Programmed Death 1; TCR, T-cell receptor; LAG3, Lymphocyte-activation gene 3; MHC, Major Histocompatibility Complex; TAA, tumor-associated antigen; TSA, tumor-specific antigen; Fab, antigen-binding fragment; Fc, fragment crystallizable region; FcR, fragment crystallizable region receptor; APC, antigen-presenting cell; DC, dendritic cell; ADC, antibody-drug conjugates; ADCC, antibody-dependent cytotoxicity; PSMA, prostate-specific membrane antigen; BiTE, Bi-specific T-cell engager; TriTE, trispecific T-cell engager; EpCAM, Epithelial cell adhesion molecule; HER2, Human Epidermal Growth Factor Receptor 2; NK, natural killer cell; NeoAgs; neoantigens; FcγRIIIA, Fc Gamma Receptor IIIa; Mφ, macrophage; NSCLC, non-small cell lung cancer. Created with BioRender.com.

Regardless of structure and design, bsAbs mechanisms of action can be summarized in immune cell engagement (ICEs), tumor associated antigen (TAA)-targeting, immune checkpoint blockade (ICBs), or Ab-drug conjugation (bsADCs) (Figure 1) [16].

#### 2.2.1. Tumor-Associated Antigens

TAA-targeting bsAbs may present the advantage of targeting more efficiently single or multiple signaling cascades. For example, ZW25 (zanidatamab) targets two different epitopes in the extracellular domains II and IV of human epidermal growth factor receptor (HER)-2. These epitopes are the same target of mAbs pertuzumab and trastuzumab, respectively [32]. JNJ-61186372 (amivantamab), inhibits epidermal growth factor receptor (EGFR) and hepatocyte growth factor receptor (HGFR; cMet) dimerization and phosphorylation, blocking signaling propagation to downstream effectors, while also promoting EGFR and MET downregulation [33]. Therefore, by dual-TAA targeting, the drug selectivity for cancer cells is enhanced, ideally reducing on target/off tumour toxicities. Moreover, the dual receptor signaling blockade could help overcome known mechanisms of drug resistance and tumor escape mechanisms.

#### 2.2.2. Antibody Drug Conjugates

Antibody drug conjugates (ADCs) are dramatically changing the landscape of cytotoxic treatments of different tumors. Compared to mAb-based ADC, bsADCs may theoretically have the advantage of improved specificity and drug internalization, with potentially enhanced tumor cell killing and potentially improved therapeutic indices [34]. The most advanced product in clinical testing is represented by ZW49, which couples N-acyl sulfonamide auristatin delivery with enhanced ZW25 internalization properties [35]. Other strategies to improve ADC activity are lysosomal shuttling enhancement, as exploited by bsHER2xCD63his (with histidine-mutated CD63) coupled to the microtubule-disrupting agent duostatin-3 [36], or the introduction of novel effective conjugates, such as toxins, radioisotopes, or cytokines [37].

#### 2.2.3. Immune Cell Engagers

ICEs are characterized by one single chain variable fragment (scFv) recognizing a surface TAA and a second scFv specific for an effector immune cells’ membrane receptor [38]. They work by redirecting immune cells against cancer cells by physically promoting a link between the two, achieving recruitment and activation of immune effector cells. On the immune cell side, ICEs work in a major histocompatibility complex (MHC)-independent manner and utilized, most commonly, CD3 (T cell engagers), followed by CD16 (natural killer, NK, cell engagers) or CD64 (phagocytic cell engagers) [25].

#### 2.2.4. Immune Checkpoint Inhibitors/Blockers

Lastly, bsAbs with specificities for immune checkpoint inhibitor/blocking (ICI, ICB) receptors exert their activity by enhancing and re-directing host’s immune responses against cancer cells [39]. While this approach could potentially result in a synergistic blockade of the inhibitory receptors, it can also lead to enhanced toxicities. One way to address these issues is, for example, by matching the right bsAb design to its specific functions. Notably, MEDI5752, a novel monovalent bsAb targeting anti-PD-1/CTLA4, was recently shown to saturate CTLA4 on PD-1 positive T cells at lower concentrations than for PD-1 negative T cells. This has been interpreted as potentially useful to decouple toxicities from anti-tumor functions. This affinity change was due to differences in valency on the CTLA4-side, linked to a reduction in potency of monovalent (i.e., MEDI5752), compared to bivalent, CTLA4 mAbs [40].

Overall, key determinants of the future positive applications of bsAbs in solid tumors involve the discovery of novel TAAs, ICB receptors or drug conjugates, the correct matching of bsAb design with its biological purpose and the clinical scenario.

## 3. State-of-the Art of Bispecific Antibodies in Solid Tumors

**Figure 2:**
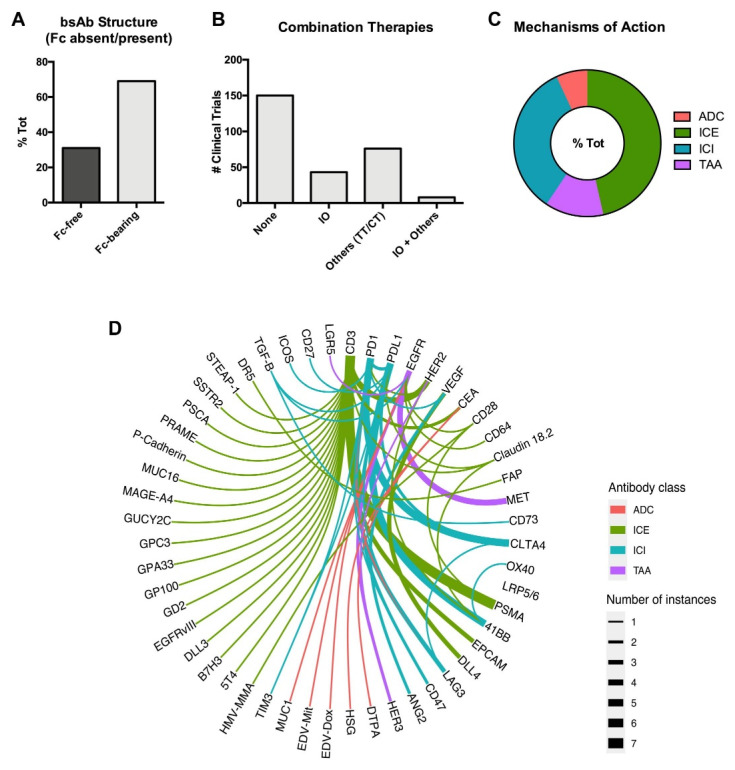
Outline of bsAb-based Clinical Trials in Solid Malignancies. (**A**) Bar graphs showing the percentage of clinical trials utilizing either Fc-free or Fc-containing bsAbs out of total number of bsAbs. Fc, Fragment, crystallizable. (**B**) Bar graphs showing the number of clinical trials utilizing bsAbs either alone or in combination with immunotherapies (IO), other agents (target therapies, TT; chemotherapies, CT), or both. (**C**) Pie chart showing the percentage of clinical trials utilizing bsAbs in solid tumors based on their mechanisms of action out of total number of bsAbs. Antibody Drug Conjugate, ADC; Immune Cell Engager, ICE; Immune Checkpoint Inhibitor, ICI; Tumor-Associated Antigen-targeting, TAA. (**D**) Chord diagram showing interactions amongst bsAbs utilized in clinical trials against solid tumors (each instance is a different drug).

**Figure 3:**
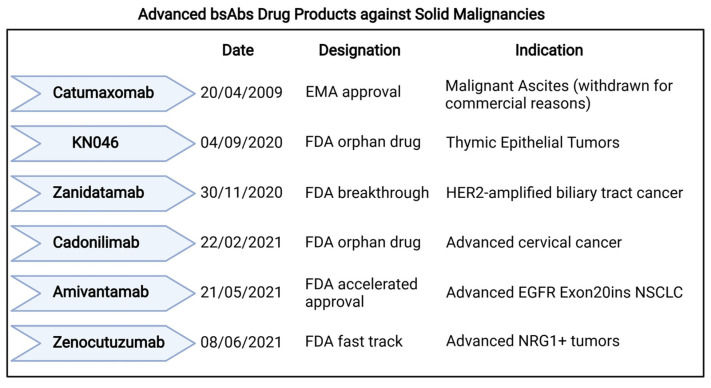
Advanced bsAbs Drug Products against Solid Malignancies. The figure depicts bsAbs receiving regulatory designations for their use in solid malignancies. Designations included, approval, accelerated approval, fast track, breakthrough, orphan drug. EMA, European Medical Agency; FDA, Food and Drug Administration; HER2, human epidermal growth factor; EGFR, Epidermal Growth Factor Receptor; NSCLC, Non-Small Cell Lung Cancer; NRG1, Neuregulin 1. Created with BioRender.com.

Although many bsAbs showed remarkable activity especially in the context of B-ALL [24], most clinical trials so far have been conducted in patients with solid malignancies, as recapitulated in Tables S1 and S2 [16]. In this setting, most trials investigated Fc-bearing bsAbs (69%) either alone (approximately 50%) or in combination with immunotherapies or other agents (i.e., target therapies or chemotherapies) (Figure 2A,B). Interestingly, most clinical trials conducted in solid tumors involve bsAbs with ICE or ICI functions (Figure 2C,D). In this section we will discuss the most promising compounds that have thus far yielded preliminary safety and, eventually, clinical results, further summarized in Figure 3.

## 4. TAA-Bispecific Targeting

Amivantamab (JNJ-61186372) is a fully-human anti-EGFR/cMET bsAb with immune cell directing activity [33,41]. While EGFR binding exploits some of the amino acids with the cetuximab binding loop (in the EGFR domain III), MET binding prevents the interaction with the hepatocyte growth factor (HGF) beta chain and subsequent downstream signaling [33]. Upon binding to its targets, amivantamab downregulates EGFR and MET membrane expression through their internalization, lysosomal degradation, and trogocytosis [42]. In addition, its Fc region shows reduced fucosylation due to low levels of fucose in the source engineered cell line, allowing for a stronger binding to fragment crystallizable gamma receptor III A (FcγRIIIA) region [43].

Amivantamab was recently granted accelerated approval by the FDA for patients affected by non-small cell lung cancer (NSCLC) harboring exon 20 insertion mutation in EGFR gene, with disease progression on/after platinum-based chemotherapy [44]. Remarkably, since available EGFR inhibitors have not demonstrated satisfying clinical activity against EGFR exon 20 mutation in NSCLC patients, amivantamab may resolve an unmet clinical need [45]. Regulatory approval was based on the preliminary results from the phase I CHRYSALIS study (NCT02609776) [46], where 81 NSCLC patients with EGFR exon 20 insertion received amivantamab once weekly for four weeks and then every two weeks until progression or unacceptable toxicity. Overall response rate (ORR) was 40% (95% CI, 29–51%) with a median duration of response (DOR) of 11.1 months (95% CI, 6.9 – NE). Adverse events (AE) included rash, infusion related reactions (IRR), musculoskeletal pain, dyspnea, fatigue, edema, and 11% of patients had to discontinue treatment due to an AE. Amivantamab was also evaluated in combination with Lazertinib in 45 chemotherapy naïve NSCLC patients with EGFR mutation (exon 19 deletion or L858R mutation) upon progression to osimertinib: ORR was obtained in 36% of patients (95% CI, 22–51%), with a median progression free survival (PFS) of 4.9 months (95% CI, 3.7–9.7), and it was associated to a manageable toxicity profile [47]. Interestingly, immunohistochemical (IHC) analysis of EGFR/MET performed on 20 patients showed high IHC scores in half of them and, among them, encouraging ORR of 90% and median PFS of 9.7 months, suggesting IHC as a possible predictive biomarker [47].

The results of CHRYSALIS study led to the design of other clinical trials. For instance, in the phase III MARIPOSA trial (NCT04487080), patients with locally-advanced or metastatic NSCLC harboring EGFR mutations (exon 19 deletion or L858R mutation) are randomized to receive either the combination of amivantamab and lazertinib, lazertinib alone or the standard of care (SoC) osimertinib alone as first-line therapy [48]. Another promising clinical trial is the ongoing phase III PAPILLON trial (NCT04538664), where amivantamab in combination with SoC (carboplatin + pemetrexed) is compared to SoC chemotherapy alone in patients with EGFR exon 20 insertion mutation positive NSCLC. The CHRYSALIS 2 trial (NCT04077463) is an ongoing phase I/Ib open-label study of lazertinib as monotherapy and in combination with amivantamab in patients with advanced EGFRm NSCLC and will also try to prospectively validate predictive IHC biomarkers identified in the CHRYSALIS trial [49].

Zanidatamab (ZW25) is a humanized biparatopic (targeting two different epitopes of the same Ag) IgG1 antibody: the HER2 extracellular domains (ECDs) ECD2 (pertuzumab binding domain) and ECD4 (trastuzumab binding domain) [50]. In November 2020, zanidatamab received the FDA breakthrough designation for previously treated HER2-amplified biliary tract cancer, based on the results of a phase I study (NCT02892123) [32]. Zanidatamab was administered intravenously every two weeks in combination with chemotherapy. AE were observed in 70% (14/21) of patients, consisting predominantly of diarrhea (43%, 9/20) and IRR (33%, 6/20). In the 17 response-evaluable patients the ORR was 47% (8/17) and the disease control rate (DCR) was 65% (11/17) [51]. The phase IIb clinical trial is currently ongoing (NCT04466891, HERIZON-BTC-01).

Zanidatamab has also been tested against HER2-high (IHC 3+ or 2+, upon testing with in situ hybridization, ISH) breast (17 patients), gastric/esophageal (11 patients), or other (5 patients) cancers with progressive disease after SoC. HER2-high breast cancer (BC) patients had received prior trastuzumab and T-DM1 (100%), pertuzumab (82%) and lapatinib (53%), with a median of six HER2-targeted regimens for metastatic disease, whereas all gastrointestinal patients had received prior trastuzumab, with a median of four lines of treatments. The DCR was 54% in the BC cohort, 57% in the gastroesophageal cohort, and 33% in other cancers [32]. In HER2-positive gastrointestinal cancer, administered alone (part 1–2) and in combination with chemotherapy (paclitaxel or capecitabine, part 3), zanidatamab demonstrated a favorable safety profile, with major AEs being diarrhea (44%) and IRR (36%) [52].

Moreover, the combination of zanidatamab plus immunotherapy is under evaluation in a phase IB/II clinical trial in first line treatment (NCT04276493): in cohort 1, patients affected by HER2+ metastatic BC will receive the drug in combination with docetaxel; in cohort 2, patients affected by HER2-positive advanced gastric/gastroesophageal junction adenocarcinoma will receive zanidatamab plus chemotherapy and tislelizumab (anti-PD1) [53]. Lastly, a phase IIa study is investigating the combination with cyclin-dependent kinase 4/6 inhibitor palbociclib and fulvestrant in advanced HER2-positive/HR-positive BC patients (NCT04224272).

KN026 is a Fc-based, biparatropic, bsAb exploiting the same specificities as trastuzumab (ECD4) and pertuzumab (ECD2). In the first in-human phase I trial (NCT03619681), no dose-limiting toxicity was observed, with an ORR of 32.1% (95% CI, 20.3–46.0) and a DCR of 76.8% (95% CI, 63.6–87.0) [54]. KN026 was also tested in HER2-positive gastric/gastroesophageal junction cancer patients failing front-line therapies, showing an ORR of 55.6% (10/18 patients) and DCR of 72.2% (13/18) in IHC3+/IHC 2+ ISH-positive patients; while ORR and DCR of 22.2% (2/9) in IHC1/2+ ISH-negative or IHC 0/1 ISH-positive patients (NCT03925974) [55]. Given the encouraging preliminary results and the favorable toxicity profile demonstrated, KN026 is also being evaluated in combination with KN046 (anti-CTLA4/PD1) bsAb (NCT04521179, NCT04040699), showing good tolerability and anti-tumor activity in preliminary analyses [56].

Zenocutuzumab (MCLA-128) is a humanized IgG1 bsAb targeting both HER2 (on a different epitope than trastuzumab) and HER3. Zenocutuzumab was designed with the purpose of overcoming the HER3-mediated resistance to HER2 target therapy by inhibiting HER2–HER3 interaction, downstream phosphoinositide 3-kinase (PI3K)-AKT signaling cascade, and by mediating ADCC activity [57].

Zenocutuzumab showed a favorable toxicity profile and promising single agent activity in heavily pre-treated patients with gastric/gastroesophageal cancers progressing to anti-HER2 inhibition [58]. Moreover, the compound is under evaluation in a phase II clinical trial, investigating the combination of this new drug with either endocrine therapy (cohort 2) or with trastuzumab (cohort 1, doublet) and vinorelbine (cohort 1, triplet). Preliminary results from cohort 1 reported a DCR of 77% (90% CI 60–89) in heavily pre-treated patients affected by HER2-positive metastatic BC progressing on TDM1. The safety profile was dominated by neutropenia (61%; 46% G3–G4), diarrhea (61%; 4% G3–G4), asthenia/fatigue (46%, all grades), and nausea (29%, all grades) [59]. In cohort 2, patients affected by ER+ HER2-low tumors received zenocutuzumab in combination with endocrine therapy after progression on a CDK4/6i. Early results demonstrated a DCR of 45% (90% CI 32–59) among 42 patients (out of 48) evaluable for efficacy. The most common severe treatment-related AEs were asthenia/fatigue (27%, 2% G3–G4), diarrhea (25%, 0% G3–G4), and nausea (21%, 0% G3–G4) [60].

Another application of zenocutuzumab is represented by metastatic solid tumors harboring Neuregulin 1 (NGR1) gene fusions [61]. Indeed, this drug prevents NRG1 fusion proteins from binding HER3, thereby avoiding HER3–HER2 interactions. Importantly, the compound was granted fast track and orphan drug designation by the FDA in 2021. Indeed, the eNRGy phase I/II basket trial (NCT02912949) and an early access program are evaluating zenocutuzumab in pancreatic, NSCLC, and other solid tumors harboring NRG1 fusion [61]. Preliminary analysis on advanced NRG1-fusion positive cancer patients (10 pancreatic, 18 NSCLC, 5 other tumors) showed a manageable safety profile and an ORR of 27% (90% CI, 15–43%), with an encouraging ORR of 40% (4/10; 90% CI, 15–70) in patients with pancreatic cancer [62].

## 5. Bispecific-Antibody Drug Conjugates

ZW49 is a zanidatamab-based bsAb conjugated, via a protease-cleavable linker, with a novel N-acyl sulfonamide auristatin payload, which blocks tubulin polymerization and cell division. Auristatin conjugation has been shown not to negatively influence HER-binding in vitro [35]. Phase I NCT03821233 trial is currently evaluating ZW49 in HER2-positive cancer patients.

TF2 is a tri-Fab bsAb, targeting Carcinoembryonic Antigen (CEA) twice and histamine-succinyl-glycine (HSG). HSG is a unique synthetic hapten, which can be labelled with a wide range of radionuclides, suitable for therapeutic or imaging purposes. Pre-targeted radioimmunotherapy (pRAIT) with TF2 exploits the radio-labelled IMP-288 binding to TF2 molecules on the cancer cells. A vast array of radionuclides can be utilized with IMP288 (i.e., 111In, 68Ga, 124I, 18F, 177Lu), as depicted in Figure 1 [63]. Such pre-targeting strategy should provide a stronger signal, while reducing the dose and toxicities [64].

## 6. Immune Cell Engagers in Solid Tumors

Cinrebafusp alfa (PRS-343) is a bispecific fusion protein targeting both HER2/4-1BB on tumour cells and T cells, respectively. Seventy patients with HER2-positive tumors received cinrebafusp alfa in a phase I clinical trial (NCT03330561) [65]. This drug was well tolerated and, for doses at/above 8 mg/kg, ORR was 40% and DCR was 70%. On this basis, a phase Ib trial (NCT03650348) was initiated to test cinrebafusp alfa in combination with atezolizumab for the treatment of HER2-positive tumors, and a phase II trial in gastric/gastroesophageal junction adenocarcinomas has been planned in combination with paclitaxel and ramucirumab.

Catumaxomab (Removab) was the first bsAb approved by the European Medicine Agency (EMA) for the treatment of solid tumors. In 2009 it was approved for the intraperitoneal treatment of malignant ascites in adults with Epithelial cell adhesion molecule (EpCAM) positive carcinomas. However, it was subsequently withdrawn for commercial reasons in 2017 [66]. Structurally, catumaxomab is a trifunctional Ab, produced with quadroma technology by a mouse IgG2a and a rat IgG2b. It binds to CD3 and EpCAM, which is expressed on the surface of epithelial cancer cells. EpCAM is a smart target for ascites, since it is expressed on tumour cells causing peritoneal carcinomatosis, while other cells of peritoneum lack EpCAM expression. The Fc portion, instead, activates NK cells, macrophage and dendritic cells [67]. The EMA approval was based on the results of a phase II/III clinical trial [68], where 258 patients with recurrent symptomatic malignant ascites, resistant to conventional treatment, were randomized to paracentesis plus intraperitoneal catumaxomab or paracentesis alone, with stratification by cancer type (129 ovarian and 129 non-ovarian). Indeed, previous studies demonstrated that catumaxomab was effective in the prevention of accumulation of peritoneal effusion and in the reduction of EpCAM positive cells in patients with ovarian cancer and malignant ascites [69]. The primary endpoint of puncture-free survival was statistically significant (46 days for catumaxomab vs. 11 days in control, HR = 0.254: *p* < 0.0001). Overall survival (OS) demonstrated a positive trend in the intervention group and was prolonged in patients with gastric cancer (71 vs. 44 days, *p* = 0.0313). However, intraperitoneal catumaxomab followed by chemotherapy failed to improve PFS and OS compared to chemotherapy alone in patients with peritoneal carcinomatosis by metastatic gastric cancer in a phase II trial [70]. Notwithstanding, intraperitoneal catumaxomab is currently under evaluation in patients with peritoneal metastasis of gastric carcinoma (NCT04222114). Despite a manageable safety profile as well (main AEs were pyrexia, abdominal pain and nausea), catumaxomab use was associated with most patients developing anti-mouse antibodies (AMA) with recurrent infusions (up to 70% patients after four cycles).

Concerning other routes of administration, systemic intravenous administration was evaluated in pre-treated patients affected by NSCLC, observing dose limiting toxicities such as G3–4 elevation of liver enzymes [71]. In addition, intravescical catumaxomab is currently being evaluated for the treatment of non-muscle invasive bladder cancer (NCT04799847, NCT04819399).

## 7. Immune Checkpoint Blockade/Inhibition

KN046 is a IgG1 bsAb targeting both PD-L1 and CTLA-4 and preventing their binding to PD1 and CD80/CD86, respectively. It is under evaluation in a Phase I clinical trial (NCT03529526) [72], where primary results on 29 patients progressing on a previous immunotherapy demonstrate a manageable safety profile, dominated mainly by pruritus (27.6%), rash (27.6%), asthenia or fatigue (20.7%); with two patients (6.9%) experiencing G3–4 treatment related AEs (anemia and IRR). ORR was 12% (3/25 evaluable patients), DCR was 52.0%. Interestingly, KN046 was also tested upfront in combination with nab-paclitaxel in metastatic triple negative breast cancer (TNBC) patients (NCT03872791). In this setting, amongst the 27 patients enrolled, the combination was well tolerated and showed preliminary median PFS of 7.33 months (4.04, NE), 12 months PFS rate of 38.3% (95% CI, 19.7–74.6%), 12 months OS of 80% (95% CI, 61.4–100%), and a median OS that was not reached in the whole population [73].

Interestingly, KN046 has been granted orphan drug designation for the treatment of patients with metastatic thymic epithelial tumors progressing on a platinum-based chemotherapy and is under evaluation in a phase II clinical trial (NCT04469725) [74]. Moreover, KN046 is currently being tested in more than ten tumor types, also in combination with KN026.

Cadonilimab (AK104) is a humanized tetravalent IgG1 bsAb targeting PD-1 and CTLA4, being granted the FDA orphan drug designation for the treatment of metastatic cervical cancer. This was due to preliminary, unpublished results obtained in a phase II for second- and third-line therapy, reporting an ORR of 47.6% and a DCR of 66.7% (NCT04380805). Cadonilimab is also under evaluation in patients affected by relapsed/refractory malignant mesothelioma (NCT03261011). Early results demonstrated an ORR of 15.4% (2/13 patients) and a DCR at eight weeks of 84.6% (11/13). A percentage of 16.7% patients experienced a grade 3–4 AEs (fever, type 1 diabetes mellitus and IRRs). Grade 1–2 AEs were mainly represented by rash and IRR.

Cadonilimab has also demonstrated a manageable safety profile with early anti-tumour activities upon combination with mXELOX chemotherapy protocol as first-line regimen for metastatic gastric/gastroesophageal junction adenocarcinoma, regardless of the PD-L1 status in the NCT03852251 phase I trial [75]. Most frequent treatment-related AEs were myelotoxicity (neutropenia, 26.5%; thrombocytopenia, 20.6%; anemia, 17.6%) and IRR (17.6%). Grade ≥ 3 AEs were represented by neutropenia (8.8%) or immune-related reactions (8.8%). ORR was 66.7% (95% CI, 44.7–84.4) with two complete responses (CR) and 14 partial responses (PR), DCR was 95.8% (95% CI, 78.9–99.9). Furthermore, in a phase II clinical trial cadonilimab was administered in combination with lenvatinib as first line treatment of unresectable hepatocellular carcinoma (BCLC stage B or C, Child Pugh class A) [76]. Out of the 18 patients evaluable for antitumor activity, early results demonstrated an ORR of 44.4% (8/18) and a DCR of 77.8%. Main AEs were represented by increased blood transaminase levels (36.7%), thrombocytopenia (33.3%), neutropenia (30.0%), and hyper-bilirubinaemia (26.7%).

## 8. Future Perspectives on Bispecific Targeting of Solid Tumors

In recent years, clinical testing of bsAb for the treatment of solid cancers has steadily grown. Some bsAbs have shown encouraging clinical response rates with favorable toxicity profiles across different patient populations. However, several factors challenge the implementation of bsAbs in solid tumors, such as limited biodistribution, microenvironmental resistance mechanisms, the emergence of anti-drug antibodies, or Ag-escape. Several tools are currently being investigated to improve bsAbs application and their efficacy in solid tumors: simultaneous multiple interaction BiTEs (SMITEs), T Cell Receptor (TCR)-armed bispecific molecules targeting tumor-specific antigens, BiTE-armed oncolytic viruses (OVs), bsAb-armed CAR-T cells, endogenous secretion of T cell redirecting Abs (STAb), or combinatorial drugs acting on the Tumor Microenvironment (TME).

SMITEs exploit the combination of two distinct BiTEs to increase specificity, augment T cell responses or convert inhibitory into stimulatory signals [28]. Interestingly, SMITEs exert their combinatorial functions at concentrations in which each single agent would not be toxic nor active. One example is the combination of an anti-CD3/TAA ICE to convert eventual inhibitory activity of a BiTE specific for CD28 [77]. Other applications of the SMITE platform may involve simultaneous multi-TAA targeting to reduce immune escape mechanisms.

Bispecific targeting of tumor cells can also be exploited by utilizing fusion proteins composed by anti-CD3 scFv coupled with a soluble, tumor-specific, TCR. Despite being burdened by MHC-restriction, this approach may allow for therapeutic targeting of large numbers of tumor- or patient-specific epitopes. A notable example is tebentafusp (IMCgp100), constituted by TAA-targeting bispecific molecules specific for CD3 and gp100 [78]. Tebentafusp has recently shown a favorable safety profile together with clinical activity in metastatic uveal melanoma in a phase I clinical trial (NCT01211262). Interestingly, tebentafusp was granted the FDA fast-track designation due to pre-planned interim analysis showing improved OS with a HR of 0.51 (95% CI, 0.36–0.71; *p* < 0.0001) compared to investigator’s choice of either dacarbazine, ipilimumab, or pembrolizumab [79]. Full phase 3 results are awaited in 2021 (NCT03070392) [80,81]. Apart from TAA-targeting, mAbs may also be designed to mimic TCRs recognizing mutation-associated neo-antigens (MANAbodies) to improve specificity [82]. Since many mutation-associated neo-antigens (MANAs) have low-expression levels, MANAbodies have been modified to increase anti-tumor responses in pre-clinical models either in the ADC or in bispecific formats [83,84]. Recently, two bispecific MANA-targeting diabodies have been tested pre-clinically, an anti-CD3/TP53mut (R175H) and an anti-KRAS G12V/Q61H-L-R [84,85].

Another tool to improve bsAb activity and reduce toxicities is to promote local expression of bsAbs within the TME. To this end, oncolytic viruses’ ability to selectively replicate within cancer cells has been exploited in preclinical models to deliver BiTEs at the tumor site. For example, the OV ICOVIR-15K has been engineered to express an EGFRxCD3 BiTE, increasing the accumulation of tumor-infiltrating T cells compared to a parental in a pre-clinical model of human lung or colorectal carcinoma [86]. Moreover, another EGFR-specific, BiTE-encoding, OV has been shown to improve survival outcomes in a mouse model of colorectal carcinoma upon combination with folate receptor α-targeted CAR-T cell therapy. Importantly, T cell activation was evidenced on both CAR-positive and negative T cells, suggesting the establishment of broad anti-tumoral immune responses [87].

In addition, another combinatorial approach envisions the use of CART.BiTEs, in which CAR-T cells also co-express and secrete functional BiTE molecules. This platform has been tested in a pre-clinical model of human glioblastoma, with T cells encoding for an anti-EGFRvIII CAR together with an anti-EGFR-CD3 BiTE, resulting in elimination of tumors displaying heterogeneous cells (EGFRvIII positive and negative), with considerable potential advantages to EGFRvIII-specific CAR-T cell therapy alone [88].

Alternatively, ex vivo gene-modified cells can be engineered to produce bsAbs endogenously, as for STAb immunotherapies [40,41,89,90]. Different cell types can be utilized: T cells, which would benefit from autocrine bsAb production at the tumor site; or scaffold-implanted mesenchymal stromal cells, which have shown anti-cancer activity at the pre-clinical level in combination with indoleamine 2,3-dioxygenase pathway inhibition [91].

Lastly, the presence of an inflamed TME, heralded by interferon-γ signaling, is linked with clinical responses to immunotherapies [92]. However, TMEs within solid tumors are often either “immune excluded”, characterized by transforming growth factor β signaling, reduced immunogenicity, and angiogenesis; or “immune desert”, with fatty acid metabolism, neuroendocrine features, and reduced immunogenicity, thus favoring resistance to immunotherapies [93]. The impact of TME on Ab-based therapies has been shown for both TAA- and ICB-targeting compounds. For example, an abundance of immune-suppressive cell populations (i.e., myeloid derived suppressor cells) has been linked to decreased response to PD-L1 blocking antibodies in TNBC patients [94]. Similarly, reduced tumor-infiltrating lymphocytes content negatively correlates with anti-HER2 targeting agents [95]. Importantly, different biomarkers that are predictive of response to immunotherapies have been proposed, such as clonal tumor mutational burden, CXCL9/CXCL12, fractalkine or CTLA4 expression, and need to be fully validated [96,97]. To improve bsAb activity in solid tumors, combinatorial strategies with TME-modulating agents or other immunotherapies are currently being investigated.

Overall, however promising, these approaches need to be clinically tested to further assess the potential role of achieving bispecific targeting for the treatment of solid cancers.

## 9. Conclusions

The deployment of bsAbs for the treatment of solid malignancies is recently experiencing a fast-paced evolution. Indeed, while increased knowledge both on cancer biology allowed for the identification of microenvironmental- or tumor-specific targets, improved drug engineering processes led to the design of bsAbs with better therapeutic indexes and/or with therapeutic cargos. Remarkable advancements in the field have been witnessed by regulatory approval of amivantamab, as well as by breakthrough designation of zanidatamab, fast-track designation for zenocutuzumab and orphan drug designations for KN046 and cadonilimab, between 2020 and 2021. Although encouraging, most preliminary results still need proper validation in large, phase II–III randomized clinical trials, when possible. Most notably, amivantamab has already changed the clinical approach to NSCLC patients harboring EGFR exon20 mutation, a population of patient devoid of any efficacious treatment regimen so far. In general, to attain positive efficacy results in solid malignancies being investigated, the identification of the most suitable bsAb molecule must match with different disease indications and with the most appropriate combination therapies. In addition, novel approaches may also promote bsAb entry in the anti-cancer armamentarium of solid tumors, such as combinations with TME-altering agents, or bsAbs delivery at the tumor site. Overall, the entire field is moving fast to finally achieve robust safety and efficacy data, potentially envisioning bsAbs as a novel treatment in the upcoming years.

## Data Availability

No new data were created or analyzed in this study. Data sharing is not applicable to this article.

## Supplementary Materials

The following are available online at https://www.mdpi.com/article/10.3390/ph14090884/s1, Table S1: Clinical Trials investigating bsAbs in Solid Malignancies. Table showing all the bsAbs in clinical trials as of June 30th 2021, after interrogation of the ClinicalTrials.gov database using the terms “bispecific”, “antibody”, “solid tumors”, “solid malignancies”, “cancer” and variations of these terms, followed by manual verification for as drug product. Table S2: Early Clinical Results of bsAbs in Solid Cancer. Table showing patient characteristics, objective clinical responses and safety parameters of the most representative clinical trials investigating bsAb agents in solid malignancies. Abbreviations: Not Available, NA; Not Estimable, NE; Not Reached, NR; Overall Response Rate, ORR; Disease Control Rate, DCR; Duration of Response, DOR; Adverse Event, AE; Infusion Related Reaction, IRR; Complete Response, CR; Partial Respose, PR; Stable Disease, SD.
