# Supplementary material for: Research and Clinical Landscape of Bispecific Antibodies for the Treatment of Solid Malignancies
Source: Pharmaceuticals (Basel). 2021 Aug 31;14(9):884. doi: 10.3390/ph14090884 (PMC8468026; doi:10.3390/ph14090884)
Supplement: Supplementary file 1 [file pharmaceuticals-14-00884-s001.zip › pharmaceuticals-1331634 - Supplementary.pdf]

pharmaceuticals

Supplementary Materials

Research and Clinical Landscape of Bispecific Antibodies for the Treatment of Solid Malignancies

Gabriele Antonarelli [1,2], Federica Giugliano [1,2], Chiara Corti [1,2], Matteo Repetto [1,2], Paolo Tarantino [1,2] and Giuseppe Curigliano [1,2,*]

[1] Division of Early Drug Development for Innovative Therapy, European Institute of Oncology, IRCCS, 20141 Milan, Italy; gabriele.antonarelli@ieo.it (G.A.); federica.giugliano@ieo.it (F.G.); chiara.corti@ieo.it (C.C.); matteo.repetto@ieo.it (M.R.); paolo.tarantino@ieo.it (P.T.)
[2] Department of Oncology and Haematology (DIPO), University of Milan, 20122 Milan, Italy
* Correspondence: giuseppe.curigliano@ieo.it; Tel.: +39-0257489599

Citation: Antonarelli, G.; Giugliano, F.; Corti, C.; Repetto, M.; Tarantino, P.; Curigliano, G. Research and Clinical Landscape of Bispecific Antibodies for the Treatment of Solid Malignancies. *Pharmaceuticals* **2021**, *14*, 884. https://doi.org/10.3390/ph14090884

Academic Editors: Yoshikatsu Koga, Hiroki Takashima and Shigehiro Koganemaru

Received: 23 July 2021
Accepted: 29 August 2021
Published: 31 August 2021

Publisher's Note: MDPI stays neutral with regard to jurisdictional claims in published maps and institutional affiliations.

Copyright: © 2021 by the authors. Submitted for possible open access publication under the terms and conditions of the Creative Commons Attribution (CC BY) license (http://creativecommons.org/licenses/by/4.0/).

	FC function present/absent	COMBO: 0; 1= IO 2 = other; 3 = IO+other	MOA 1= ICE; 2 = TAA; 3 = ICI; 4 = ADC	Target 1	Target 2	NCT Number	Title	Status	Conditions	Sponsor/Collaborators	Phases	Enrollment	Platform
**LY3434172**	0	0	3	PD1	PDL1	NCT03936959	A Study of LY3434172, a PD-1 and PD-L1 Bispecific Antibody, in Advanced Cancer	Active, not recruiting	Advanced Cancer	Eli Lilly and Company	Phase 1	40	KIH
**LY3415244**	na	0	3	PDL1	TIM3	NCT03752177	A Study of LY3415244 in Participants With Advanced Solid Tumors	Terminated	Solid Tumor	Eli Lilly and Company	Phase 1	12	na
**LY3164530**	1	0	2	EGFR	MET	NCT02221882	A Study of LY3164530 in Participants With Cancer	Completed	Neoplasms | Neoplasm Metastasis	Eli Lilly and Company	Phase 1	29	na
**Zenocutuzumab (MCLA-128)**	1	0	2	HER2	HER3	NCT04100694	Early Access Program Providing HER2/HER3 Bispecific Antibody, MCLA-128, for a Patient With Advanced NRG1-Fusion Positive Solid Tumor	Available	NRG1 | NRG 1 Fusion | Pancreatic Cancer | Non Small Cell Lung Cancer | Solid Tumor, Unspecified, Adult	Merus N.V.			DEEK
		0				NCT02912949	A Study of Zenocutuzumab (MCLA-128) in Patients With Solid Tumors Harboring an NRG1 Fusion	Recruiting	Solid Tumours Harboring NRG1 Fusion | NSCLC Harboring NRG1 Fusion | Pancreatic Cancer Harboring NRG1 Fusion | NRG1 Fusion	Merus N.V.	Phase 1 | Phase 2	250	
		2				NCT03321981	MCLA-128 With Trastuzumab/Chemotherapy in HER2+ and With Endocrine Therapy in ER+ and Low HER2 Breast Cancer	Active, not recruiting	Breast Cancer Metastatic	Merus N.V.	Phase 2	101	
**MCLA-129**	1	0	2	EGFR	MET	NCT04930432	Study of MCLA-129, a Human Bispecific EGFR and cMet	Not yet recruiting	Solid Tumor, Non-Small Cell Lung Cancer, Head and	Betta Pharmaceuticals Co., Ltd.	Phase 1 | Phase 2	400	Biclonics

							Antibody, in Patients With Advanced NSCLC and Other Solid Tumors		Neck Cancer, Colorectal Cancer				
		0				NCT04868877	A Phase 1/2 Study Evaluating MCLA-129, a Human Anti-EGFR and Anti-c-MET Bispecific Antibody, in Patients With Advanced NSCLC and Other Solid Tumors	Recruiting	Non-Small Cell Lung Cancer Metastatic|Gastric Cancer|Head and Neck Cancer	Merus N.V.	Phase 1|Phase 2	150	
**MCLA-145**	1	0	3	PDL1	41BB	NCT03922204	A Study of Bispecific Antibody MCLA-145 in Patients With Advanced or Metastatic Malignancies	Recruiting	Advanced Cancer|Solid Tumor, Adult|B-cell Lymphoma, Adult	Merus N.V.|Incyte Corporation	Phase 1	118	Biclonics
**MCLA-158**	1	0	2	EGFR	LGR5	NCT03526835	A Study of Bispecific Antibody MCLA-158 in Patients With Advanced Solid Tumors	Unknown status	Advanced/Metastatic Solid Tumors|Colorectal Cancer	Merus N.V.|Chiltern International Inc.|Q2 Solutions|Oncology Therapeutic Development (OTD)|4Clinics	Phase 1	120	Biclonics
**Cadonilimab (AK104)**	1	0	3	PD1	CTLA4	NCT04172454	Safety and Efficacy of AK104, a PD-1/CTLA-4 Bispecific Antibody, in Selected Advanced Solid Tumors	Not yet recruiting	Advaced Solid Tumors|Melanoma	Akeso|Akeso Pharmaceuticals, Inc.	Phase 1|Phase 2	120	ITab
		2				NCT03852251	A Study of AK104, a PD-1/CTLA-4 Bispecific Antibody, for Advanced Solid Tumors or With mXELOX as First-line Therapy for Advanced Gastric or GEJ Adenocarcinoma	Unknown status	Gastric Adenocarcinoma|Advanced Solid Tumors|Gastroesophageal Junction Adenocarcinoma	Akeso|Akeso Pharmaceuticals, Inc.	Phase 1|Phase 2	112	
		0				NCT04556253	AK104 in Locally Advanced MSI-H/dMMR Gastric Carcinoma and Colorectal Cancer	Not yet recruiting	MSI-H/dMMR Gastric Carcinoma and Colorectal Cancer	Peking University	Phase 2	29	
		0				NCT04380805	A Study of AK104, a PD-1/CTLA-4	Recruiting	Recurrent Cervical	Akeso|Akesobio Australia Pty Ltd	Phase 2	40	

		Bispecific Antibody in Subjects With Recurrent/Metastatic Cervical Cancer		Cancer|Metastatic Cervical Cancer			
0	NCT04547101	A Study of AK104 in Subjects With Locally Advanced Unresectable or Metastatic MSI-H/dMMR Solid Tumors	Recruiting	MSI-H/dMMR Solid Tumor	Akeso|Akeso Pharmaceuticals, Inc.	Phase 2	70
0	NCT04220307	A Study of a PD-1/CTLA-4 Bispecific Antibody AK104 in Patients With Metastatic Nasopharyngeal Carcinoma	Not yet recruiting	Nasopharyngeal Carcinoma	Akeso|Akeso Pharmaceuticals, Inc.	Phase 2	140
2	NCT04868708	A Study of AK104 an Anti-PD-1 and Anti-CTLA-4 Bispecific Antibody) in Recurrent or Metastatic Cervical Cancer	Not yet recruiting	Recurrent or Metastatic Cervical Cancer	Akeso	Phase 2	60
2	NCT04646330	A Trial of AK104 Plus Anlotinib in NSCLC	Not yet recruiting	NSCLC	Akeso	Phase 1|Phase 2	120
2	NCT04572152	A Study of AK119 (Anti-CD73) in Combination With AK104 in Subjects With Advanced Solid Tumors	Not yet recruiting	Advanced or Metastatic Solid Tumors	Akeso	Phase 1	195
1	NCT03261011	Study of the Safety, Pharmacokinetics, and Antitumor Activity of AK104 in Subjects With Advanced Solid Tumors	Unknown status	Advanced Cancer	Akesobio Australia Pty Ltd|Akeso	Phase 1	153
0	NCT04647344	A Study of AK104 in Patients With Locally Advanced or Metastatic Non-small Cell Lung Cancer	Not yet recruiting	Lung Cancer Non-Small Cell Stage IIIB/IIIC/IV	Akeso	Phase 1|Phase 2	60
2	NCT04544644	A Study of Combination Therapy in NSCLC	Not yet recruiting	Non-small Cell Lung Cancer	Chinese PLA General Hospital	Phase 2	30

		2				NCT04728321	A Study of Anti-PD-1/CTLA-4 Bispecific AK104 Alone or in Combination With Lenvatinib in Advanced Hepatocellular Carcinoma	Recruiting	Hepatocellular Carcinoma	Akeso∣Akeso Pharmaceuticals, Inc.	Phase 2	75	
		2				NCT04444167	A Study of Anti-PD-1/CTLA-4 Bispecific AK104 Plus Lenvatinib in First-line Advanced Hepatocellular Carcinoma	Recruiting	Hepatocellular Carcinoma	Akeso∣Akeso Pharmaceuticals, Inc.	Phase 1∣Phase 2	30	
**AK112**	1	0	3	PD1	VEGF	NCT04047290	A Study of AK112, a PD-1/VEGF Bispecific Antibody, for Advanced Solid Tumors	Not yet recruiting	Neoplasms Malignant	Akesobio Australia Pty Ltd∣Akeso	Phase 1	132	na
		0				NCT04900363	A Trial of AK112 (PD-1/VEGF Bispecific Antibody) in Patients With NSCLC	Recruiting	Non-small Cell Lung Cancer	Akeso	Phase 1∣Phase 2	360	
		3				NCT04736823	A Trial of AK112 (PD1/VEGF Bispecific) in Combination With Chemotherapy in Patients With NSCLC	Recruiting	NSCLC	Akeso	Phase 2	206	
		0				NCT04597541	A Study of AK112, a PD-1/VEGF Bispecific Antibody, for Advanced Solid Tumors	Not yet recruiting	Solid Tumor, Adult	Akeso	Phase 1∣Phase 2	264	
		0				NCT04870177	Study of AK112 in the Treatment of Advanced Gynecological Tumors	Recruiting	Gynecologic Cancer∣Cancer Metastatic∣Ovarian Neoplasms∣Cervical Neoplasm∣Endometrial Neoplasms	Akeso	Phase 2	270	

**TF2**	0	2	4	CEA	HSG	NCT00895323	Bispecific Antibody in Finding Tumor Cells in Patients With Colorectal Cancer	Unknown status	Colorectal Cancer	Garden State Cancer Center at the Center for Molecular Medicine and Immunology∣National Cancer Institute (NCI)	Phase 1	9	Dock-and-Load
		2				NCT00860860	Study of Pretargeted Radioimmunotherapy of a Anti-CEA Bispecific Antibody and Lu177-labeled Peptide in Colorectal Cancer	Completed	Colorectal Neoplasms	Radboud University	Phase 1	20	
		2				NCT01730612	ImmunoTEP au 68-Ga- IMP-288 for Patients With a Recurrence of HER2 Negative Breast Carcinoma Expressing CEA	Completed	HER2 Negative Breast Carcinoma Expressing CEA	Nantes University Hospital∣Institut National de la SantÃ© Et de la Recherche MÃ©dicale, France∣Gilead Sciences	Phase 1∣Phase 2	23	
		2				NCT01273402	Study of TF2 Carcinoembryonic Antigen (CEA) Antibody in Patients With Metastatic Colorectal Cancer	Withdrawn	Metastatic Colorectal Cancer	Gilead Sciences∣National Cancer Institute (NCI)	Phase 1	0	
		2				NCT01221675	TF2- Small Cell Lung Cancer Radio Immunotherapy	Completed	Small Cell Lung Cancer∣CEA-expressing Non Small Cell Lung Carcinoma (NSCLC)	Centre RenÃ© Gauducheau	Phase 1∣Phase 2	18	
**GEM3PSCA**	0	0	1	CD3	PSCA	NCT03927573	Study With Bispecific Antibody Engaging T-cells, in Patients With Progressive Cancer Diseases With Positive PSCA Marker	Recruiting	Non-small Cell Lung Cancer∣Breast Cancer∣Pancreatic Cancer∣Prostate Cancer∣Renal Cancer∣Transitional Cell Carcinoma	GEMoaB Monoclonals GmbH∣GCP-Service International Ltd. & Co. KG	Phase 1	24	na
**Nivatrotamab (Hu3F8-BsAb)**	1	0	1	CD3	GD2	NCT03860207	Study of the Safety and Efficacy of Humanized 3F8 Bispecific Antibody (Hu3F8-BsAb) in Patients With	Recruiting	Neuroblastoma∣Osteosarcoma∣Other Solid Tumor Cancers	Y-mAbs Therapeutics	Phase 1∣Phase 2	30	Y-mAbs

							Relapsed/Refractory Neuroblastoma, Osteosarcoma and Other Solid Tumor Cancers						
		0				NCT04750239	Safety and Clinical Activity of Nivatrotamab in Relapsed/Recurrent Metastatic Small-cell Lung Cancer	Not yet recruiting	SCLC	Y-mAbs Therapeutics	Phase 1|Phase 2	69	
**MDX447**	0	2	1	CD64	EGFR	NCT00005813	Bispecific Antibody Plus White Blood Cells in Treating Patients With Recurrent or Refractory Glioblastoma Multiforme	Completed	Brain and Central Nervous System Tumors	Dartmouth-Hitchcock Medical Center|National Cancer Institute (NCI)	Phase 1		na
		2				NCT02665416	Study Evaluating the Safety, Pharmacokinetics (PK), Pharmacodynamics (PD), and Therapeutic Activity of Selicrelumab (RO7009789) With Vanucizumab or Bevacizumab in Participants With Metastatic Solid Tumors	Completed	Advanced/Metastatic Solid Tumors	Hoffmann-La Roche	Phase 1	94	
**RO5520985 (Vanucizumab)**	1	1	3	VEGF	Ang2	NCT01688206	A Study of Vanucizumab (RO5520985) Alone or in Combination With Atezolizumab in Participants With Locally Advanced or Metastatic Solid Tumors	Completed	Neoplasms	Hoffmann-La Roche	Phase 1	132	CrossMab
		2				NCT02715531	A Study of the Safety and Efficacy of Atezolizumab Administered in Combination With Bevacizumab and/or Other Treatments in Participants With Solid Tumors	Active, not recruiting	Solid Tumor	Hoffmann-La Roche	Phase 1	430	
		2				NCT02141295	A Study Comparing the	Terminated	Colorectal Cancer	Hoffmann-La Roche	Phase 2	197	

							Efficacy and Safety of Vanucizumab and FOLFOX With Bevacizumab and FOLFOX in Participants With Untreated Metastatic Colorectal Cancer						
**RO7247669 (RG6139)**	1	2	3	PD1	LAG3	NCT04524871	A Study Evaluating the Efficacy and Safety of Multiple Immunotherapy-Based Treatment Combinations in Patients With Advanced Liver Cancers (Morpheus-Liver)	Recruiting	Advanced Liver Cancers	Hoffmann-La Roche	Phase 1 | Phase 2	280	CrossMab
		0				NCT04140500	Dose Escalation Study of a PD1-LAG3 Bispecific Antibody in Patients With Advanced and/or Metastatic Solid Tumors	Recruiting	Solid Tumors | Metastatic Melanoma | Non-small Cell Lung Cancer | Esophageal Squamous Cell Carcinoma	Hoffmann-La Roche	Phase 1	320	
**RO6874813 (RG7386)**	1	0	1	FAP	DR5	NCT02558140	A Dose Escalation Study of RO6874813 in Participants With Locally Advanced or Metastatic Solid Tumors	Completed		Hoffmann-La Roche	Phase 1	120	CrossMab
**MEHD7945 A (RG7597, Duligotumab)**	1	2	2	EGFR	HER3	NCT01986166	A Study of MEHD7945A and Cobimetinib in Patients With Locally Advanced or Metastatic Cancers With Mutant KRAS	Completed	Neoplasms	Genentech, Inc.	Phase 1	23	CrossMab
		0				NCT01207323	A Study of the Safety and Pharmacokinetics (PK) of MEHD7945A in Participants With Locally Advanced or Metastatic Epithelial Tumors	Completed	Epithelial Tumors, Malignant	Genentech, Inc.	Phase 1	66	
		2				NCT01911598	A Study of MEHD7945A in Combination With Cisplatin	Completed	Head and Neck Cancer	Genentech, Inc.	Phase 1	24	

							and 5-Fluorouracil (5-FU) or Paclitaxel and Carboplatin in Participants With Recurrent/Metastatic Squamous Cell Carcinoma of the Head and Neck (R/M SCCHN)						
		2				NCT01652482	Safety and Efficacy Study of MEHD7945A + FOLFIRI Versus Cetuximab + FOLFIRI as Second Line Therapy in Participants With KRAS Wild-Type Metastatic Colorectal Cancer (mCRC)	Completed	Colorectal Cancer	Genentech, Inc.	Phase 2	135	
		2				NCT01577173	A Study of MEHD7945A Versus Cetuximab in Patients With Recurrent/Metastatic Squamous Cell Carcinoma of The Head And Neck	Completed	Head and Neck Cancer	Genentech, Inc.	Phase 2	122	
**RO7121661 (RG7769)**	1	0	3	PD1	TIM3	NCT03869190	Study Evaluating the Efficacy and Safety of Multiple Immunotherapy-Based Treatments and Combinations in Patients With Urothelial Carcinoma (MORPHEUS-UC)	Recruiting	Urothelial Carcinoma Bladder Cancer	Hoffmann-La Roche	Phase 1|Phase 2	735	CrossMab/KIH
		0				NCT03708328	A Dose Escalation and Expansion Study of RO7121661, a PD-1/TIM-3 Bispecific Antibody, in Participants With Advanced and/or Metastatic Solid Tumors	Recruiting	Solid Tumors|Metastatic Melanoma|Non-small Cell Lung Cancer (NSCLC)|Small Cell Lung Cancer (SCLC)|Esophageal Squamous Cell Carcinoma (ESCC)	Hoffmann-La Roche	Phase 1	280	
**RO6958688 (RG7802,**	1	2	1	CD3	CEA	NCT02324257	A Study of RO6958688 in	Completed	Solid Tumors	Hoffmann-La Roche	Phase 1	149	CrossMab

**Cibisatamab)**			Participants With Locally Advanced and/or Metastatic Carcinoembryonic Antigen Positive Solid Tumors					
	1	NCT02650713	A Study of the Safety, Pharmacokinetics, and Therapeutic Activity of RO6958688 in Combination With Atezolizumab in Participants With Locally Advanced and/or Metastatic Carcinoembryonic Antigen (CEA)-Positive Solid Tumors	Completed	Solid Tumors	Hoffmann-La Roche	Phase 1	228
	1	NCT04826003	Study To Evaluate Safety, Pharmacokinetics, Pharmacodynamics, And Preliminary Anti-Tumor Activity Of RO7122290 In Combination With Cibisatamab With Obinutuzumab Pre-Treatment	Recruiting	Metastatic Colorectal Cancer	Hoffmann-La Roche	Phase 1|Phase 2	80
	1	NCT03337698	A Study Of Multiple Immunotherapy-Based Treatment Combinations In Participants With Metastatic Non-Small Cell Lung Cancer (Morpheus- Non-Small Cell Lung Cancer)	Recruiting	Carcinoma, Non-Small-Cell Lung	Hoffmann-La Roche	Phase 1|Phase 2	380
	1	NCT03866239	A Phase Ib Study to Evaluate the Safety, Efficacy, and Pharmacokinetics of Cibisatamab in Combination With Atezolizumab After	Recruiting	Colorectal Cancer	Hoffmann-La Roche	Phase 1	46

							Pretreatment With Obinutuzumab in Participants With Previously Treated Metastatic Colorectal Adenocarcinoma						
**RO7121661 + RO7247669**	1	1	4	PD1	TIM3/LAG3	NCT04785820	A Study of RO7121661 and RO7247669 Compared With Nivolumab in Participants With Advanced or Metastatic Squamous Cell Carcinoma of the Esophagus	Recruiting	Advanced or Metastatic Esophageal Squamous Cell Carcinoma	Hoffmann-La Roche	Phase 2	255	CrossMab
**CDX-527**	1	0	3	PDL1	CD27	NCT04440943	A Study of the PD-L1xCD27 Bispecific Antibody CDX-527 in Patients With Advanced Malignancies	Recruiting	Non-small Cell Lung Cancer∣Breast Cancer∣Gastric Cancer∣Renal Cell Carcinoma∣Ovarian Cancer∣Primary Peritoneal Carcinoma∣Fallopian Tube Cancer∣Cholangiocarcinoma∣Bladder Urothelial Carcinoma∣MSI-H Colorectal Cancer∣Esophageal Cancer∣Hepatic Cancer∣Head and Neck Cancer∣Other Solid Tumors	Celldex Therapeutics	Phase 1	40	na
**SI-B001**	1	0	2	EGFR	HER3	NCT04603287	A Study of SI-B001, an EGFR/HER3 Bispecific Antibody, in Locally Advanced or Metastatic Epithelial Tumors	Recruiting	Locally Advanced or Metastatic Epithelial Tumor	Sichuan Baili Pharmaceutical Co., Ltd.∣SystImmune Inc.	Phase 1	96	na

**SI-B003**	na	0	3	PD1	CTLA4	NCT04606472	A Study of SI-B003, a PD-1/CTLA-4 Bispecific Antibody, in Patients With Advanced Solid Tumors+H27	Recruiting	Solid Tumor	Sichuan Baili Pharmaceutical Co., Ltd.丨SystImmune Inc.	Phase 1	159	na
**ZW25 (Zanidatamab)**	1	1	2	HER2	HER2	NCT04276493	Anti-HER2 Bispecific Antibody ZW25 Activity in Combination With Chemotherapy With/Without Tislelizumab	Recruiting	Breast Cancer丨Gastric Cancer丨Gastroesophageal Junction Cancer	BeiGene	Phase 1丨Phase 2	50	Azymetric
		0				NCT04466891	A Study of ZW25 (Zanidatamab) in Subjects With Advanced or Metastatic HER2-Amplified Biliary Tract Cancers	Recruiting	HER2-amplified Biliary Tract Cancers	Zymeworks Inc.丨BeiGene, Ltd.	Phase 2	100	
		0				NCT04513665	ZW25 in Women With Endometrial Cancers	Recruiting	Endometrial Cancer, Carcinosarcoma	Zymeworks Inc.	Phase 2	25	
		2				NCT03929666	A Safety and Efficacy Study of ZW25 (Zanidatamab) Plus Combination Chemotherapy in HER2-expressing Gastrointestinal Cancers, Including Gastroesophageal Adenocarcinoma, Biliary Tract Cancer, and Colorectal Cancer	Recruiting	HER2-expressing Gastrointestinal Cancers, Including Gastroesophageal Adenocarcinoma, Biliary Tract Cancer, and Colorectal Cancer	Zymeworks Inc.	Phase 2	362	
		2				NCT02892123	Trial of ZW25 (Zanidatamab) in Patients With Advanced HER2-expressing Cancers	Recruiting	HER2-expressing Cancers	Zymeworks Inc.	Phase 1	280	
		2				NCT04224272	A Study of ZW25 (Zanidatamab) With Palbociclib Plus Fulvestrant in Patients With HER2+/HR+ Advanced Breast Cancer	Recruiting	HER2+/HR+ Breast Cancer	Zymeworks Inc.	Phase 2	86	

**ZW49**	1	2	4	HER2	HER2	NCT03821233	A Dose Finding Study of ZW49 in Patients With HER2-Positive Cancers	Recruiting	HER2-expressing Cancers	Zymeworks Inc.	Phase 1	174	Azymetric
**TNB-585**	1	0	1	CD3	PSMA	NCT04740034	A Study of TNB-585 in Subjects With Metastatic Castrate-Resistant Prostate Carcinoma	Recruiting	Metastatic Castration-resistant Prostate Cancer	Teneobio, Inc.	Phase 1	72	na
**rM28**	0	0	1	CD28	HMV-MAA	NCT00204594	Local Treatment of Metastatic Melanoma With Autologous Lymphocytes and the Bispecific Antibody rM28	Completed	Malignant Melanoma	University Hospital Tuebingen	Phase 1|Phase 2	1	na
**INBRX-105 (ES101)**	1	0	3	PDL1	41BB	NCT04009460	A Study of ES101 (PD-L1x4-1BB Bispecific Antibody) in Patients With Advanced Solid Tumors	Recruiting	Solid Tumors|Neoplasms|Malignant Tumor	Elpiscience Biopharma, Ltd.	Phase 1	180	na
		0				NCT04841538	A Study of ES101 (PD-L1x4-1BB Bispecific Antibody) in Patients With Advanced Malignant Thoracic Tumors	Not yet recruiting	Thoracic Tumors|Non-small Cell Lung Cancer|Small Cell Lung Cancer	Elpiscience Biopharma, Ltd.	Phase 1|Phase 2	276	
		0				NCT03809624	Study of INBRX-105 in Patients With Solid Tumors	Recruiting	Metastatic Solid Tumors|Non-small Cell Lung Cancer|Melanoma|Head and Neck Squamous Cell Carcinoma|Gastric Adenocarcinoma|Renal Cell Carcinoma|Urothelial Carcinoma|Esophageal Adenocarcinoma	Inhibrx, Inc.	Phase 1	90	
**Catumaxomab (Removab)**	1	0	1	CD3	EpCAM	NCT00189345	Randomized, Multicenter, 2-Dose Level. Open-Label, Phase IIa Study With the	Completed	Ovarian Cancer|Fallopian Tube	AGO Study Group	Phase 2	44	Triomab

		Intraperitoneally Infused Trifunctional Bispecific Antibody Removab(TM) (Anti-EpCAM x Anti-CD3) to Select the Better Dose Level in Platinum Refractory Epithelial Ovarian Cancer Patients		Neoplasms|Peritoneal Neoplasms			
0	NCT00464893	Phase II Study With Catumaxomab in Patients With Gastric Cancer After Neoadjuvant CTx and Curative Resection	Completed	Gastric Cancer|Gastric Adenocarcinoma	Neovii Biotech	Phase 2	70
0	NCT00836654	Study in EpCAM Positive Patients With Symptomatic Malignant Ascites Using Removab Versus an Untreated Control Group	Completed	EpCam Positive Tumor (e.g.Ovarian, Gastric, Colon, Breast)|Malignant Ascites	Neovii Biotech	Phase 2|Phase 3	258
0	NCT00352833	Safety and Efficacy Study With Catumaxomab in Patients After Curative Resection of a Gastric Adenocarcinoma	Completed	Gastric Cancer|Gastric Adenocarcinoma	Neovii Biotech	Phase 2	40
0	NCT04819399	Investigation of Safety and Tolerability of Catumaxomab in Patients With NMIBC	Recruiting	Urinary Bladder Neoplasms	Lindis Biotech GmbH	Phase 1	30
0	NCT00377429	Safety and Efficacy Study of Catumaxomab to Treat Ovarian Cancer After a Complete Response to Chemotherapy	Completed	Ovarian Cancer	Neovii Biotech|Fresenius Biotech North America	Phase 2	47
2	NCT01815528	Feasibility and Clinical Activity of Initial Intraperitoneal Catumaxomab Followed by Chemotherapy in Patients With	Completed	Recurrent Epithelial Ovarian Cancer	JSehouli|Charite University, Berlin, Germany	Phase 2	2

		Recurrent Ovarian Cancer					
0	NCT01065246	Safety Study of Second Intraperitoneal (I.P.) Infusion Cycle of Catumaxomab in Patients With Malignant Ascites	Completed	Malignant Ascites Due to Epithelial Carcinoma	Neovii Biotech	Phase 2	8
0	NCT00326885	Study of the Trifunctional Antibody Catumaxomab to Treat Recurrent Symptomatic Malignant Ascites	Completed	Malignant Ascites	Neovii Biotech | Fresenius Biotech North America	Phase 2	32
0	NCT01246440	Catumaxomab as a Consolidation Therapy in Patients With Ovarian Cancer in Second or Third Clinical Disease Remission	Completed	Ovarian Cancer	Grupo Espa√±ol de Investigaci√≥n en C√°ncer de Ovario | Neovii Biotech	Phase 2	39
0	NCT01504256	Catumaxomab for Treatment of Peritoneal Carcinomatosis in Patients With Gastric Adenocarcinomas	Completed	Gastric Adenocarcinoma With Peritoneal Carcinomatosis | Siewert Type II Adenocarcinoma of Esophagogastric Junction With Peritoneal Carcinomatosis | Siewert Type III Adenocarcinoma of Esophagogastric Junction With Peritoneal Carcinomatosis	AIO-Studien-gGmbH | Neovii Biotech	Phase 2	42
0	NCT01320020	A Open Label, Dose Escalating Study to Evaluate the Safety and Tolerability of Ascending Intravenous (i.v.) Doses of Catumaxomab in Epithelial Cancer Patients	Terminated	Epithelial Cancer Patients	Neovii Biotech	Phase 1	16

0	NCT00822809	CASIMAS: Catumaxomab Safety Phase IIIb Study With Intraperitoneal Infusion in Patients With Malignant Ascites Due to Epithelial Cancers	Completed	Cancer|Neoplasms|Carcinoma|Malignant Ascites	Neovii Biotech	Phase 3	230
0	NCT00563836	Phase II Study of the Trifunctional Antibody Catumaxomab Administered Intra- and Post-operatively in Patients With Ovarian Cancer	Completed	Ovarian Cancer|Epithelial Ovarian Cancer	Neovii Biotech	Phase 2	41
0	NCT01784900	Treatment of Gastric Peritoneal Carcinomatosis by Association of Complete Surgical Resection of the Lesions and Intraperitoneal Immunotherapy Using Catumaxomab	Terminated	Patients With Gastric Peritoneal Carcinomatosis	Gustave Roussy, Cancer Campus, Grand Paris	Phase 2	26
0	NCT04222114	Comparing the Efficacy and Safety of Intraperitoneal Infusion of Catumaxomab and Treatment of Investigator Choice in Patients With Advanced Gastric Carcinoma With Peritoneal Metastasis	Recruiting	Malignant Ascites|Gastrointestinal Cancers	LintonPharm Co.,Ltd.	Phase 3	282
0	NCT04799847	A Multicenter, Non-randomized, Uncontrolled, Open-label Phase I/II Study to Observe the Safety and Preliminary Efficacy of Catumaxomab in Patients With Non-Muscle-Invasive Bladder Cancer Who Have Failed or Are Intolerant to BCG Vaccine	Recruiting	Bladder Cancer	LintonPharm Co.,Ltd.	Phase 1|Phase 2	167

**EMB-01**	1	0	2	EGFR	cMET	NCT03797391	A Dose Escalation Study of EMB-01 in Participants With Advanced/Metastatic Solid Tumors	Recruiting	Neoplasms|Neoplasm Metastasis|Non-Small-Cell Lung Cancer	Shanghai EpimAb Biotherapeutics Co., Ltd.|Covance	Phase 1|Phase 2	73	FIT-Ig
**EMB-02**	1	0	3	PD1	LAG3	NCT04618393	A Study of EMB-02 in Participants With Advanced Solid Tumors	Recruiting	Advanced Solid Tumors	Shanghai EpimAb Biotherapeutics Co., Ltd.	Phase 1|Phase 2	43	na
**hMN-14 × m734**	0	2	4	CEA	DTPA	NCT00467506	Phase II Two-step Radioimmunotherapy Clinical Study in Medullary Thyroid Carcinoma	Completed	Thyroid Neoplasms	Nantes University Hospital	Phase 2		na
**HX009**	1	0	3	PD1	CD47	NCT04097769	The Safety, Tolerability, and Initial Efficacy of HX009 in Patients With Advanced Malignancies	Recruiting	Advaced Solid Tumors	Waterstone Hanxbio Pty Ltd	Phase 1	37	na
		0				NCT04886271	Recombinant Humanized Anti-CD47 / PD-1 Bifunctional Antibody HX009 Injection in the Treatment of Advanced Solid Tumors	Recruiting	Advanced Solid Tumor	Waterstone Hanxbio Pty Ltd	Phase 2	210	
**FS118**	0	0	3	PDL1	LAG3	NCT03440437	FS118 First in Human Study in Patients With Advanced Malignancies	Recruiting	Advanced Cancer|Metastatic Cancer|Squamous Cell Carcinoma of Head and Neck	F-star Delta Limited	Phase 1|Phase 2	80	na
**FS120**	0	0	3	OX40	41BB	NCT04648202	FS120 First in Human Study in Patients With Advanced Malignancies	Recruiting	Advanced Cancer|Metastatic Cancer	F-star Beta Limited	Phase 1	70	na
**FS222**	1	0	3	PDL1	41BB	NCT04740424	FS222 First in Human Study in Patients With Advanced Malignancies	Recruiting	Advanced Cancer|Metastatic Cancer	F-star Beta Limited	Phase 1	177	na
**PF-06671008**	0	0	1	CD3	P-Cadherin	NCT02659631	PF-06671008 Dose Escalation Study in Advanced Solid Tumors	Terminated		Pfizer	Phase 1	28	DART/KIH
**PF-07062119**	1	1	1	CD3	GUCY2C	NCT04171141	Study to Test the Safety and Tolerability of	Recruiting	Gastrointestinal Tumors,	Pfizer	Phase 1	130	na

							PF-07062119 in Patients With Selected Advanced or Metastatic Gastrointestinal Tumors		Colorectal Adenocarcinomas, Gastric Adenocarcinomas, Esophageal Adenocarcinomas				
**PF-07257876**	na	0	3	PDL1	CD47	NCT04881045	Study to Test the Safety and Tolerability of PF-07257876 in Participants With Selected Advanced Tumors.	Not yet recruiting	Non-Small Cell Lung Cancer|Squamous Cell Carcinoma of the Head and Neck|Ovarian Cancer	Pfizer	Phase 1	90	na
**AGEN1223**	1	1	3	na	na	NCT04156100	A Study in Subjects With Advanced Solid Tumors	Active, not recruiting	Advanced Solid Tumor	Agenus Inc.	Phase 1	82	na
**GEN1044**	1	0	1	CD3	5T4	NCT04424641	A Study on the Safety of GEN1044 (DuoBody CD3x5T4) in Subjects With Malignant Solid Tumors	Recruiting	Dose Escalation Part: Locally Advanced or Metastatic Solid Tumor(s)|Expansion Part: Prostate Cancer|Expansion Part: Esophageal Cancer|Expansion Part: Triple Negative Breast Cancer (TNBC)|Expansion Part: Squamous Cell Carcinoma of Head and Neck (SCCHN)|Expansion Part: Non-small Cell Lung Cancer (NSCLC)|Expansion Part: Bladder Cancer|Expansion Part: Uterine Cancer	Genmab|AbbVie	Phase 1|Phase 2	378	na
**REGN4018**	1	1	1	CD3	MUC16	NCT04590326	Study of REGN5668 Administered in Combination With	Recruiting	Ovarian CancerFallopian Tube Cancer-Primary	Regeneron Pharmaceuticals	Phase 1|Phase 2	290	na

							Cemiplimab or REGN4018 in Adult Women With Recurrent Ovarian Cancer.		Peritoneal Cancer				
		1				NCT03564340	Study of REGN4018 Administered Alone or in Combination With Cemiplimab in Patients With Recurrent Ovarian Cancer	Recruiting	Recurrent Ovarian Cancer|Recurrent Fallopian Tube Cancer|Recurrent Primary Peritoneal Cancer	Regeneron Pharmaceuticals	Phase 1|Phase 2	392	
**REGN5093**	1	0	1	MET	MET	NCT04077099	REGN5093 in Patients With MET-Altered Advanced Non-Small Cell Lung Cancer	Recruiting	NSCLC	Regeneron Pharmaceuticals	Phase 1|Phase 2	111	na
**REGN5678**	1	1	1	CD28	PSMA	NCT03972657	Study of REGN5678 (Anti-PSMAxCD28) With Cemiplimab (Anti-PD-1) in Patients With Metastatic Castration-resistant Prostate Cancer	Recruiting	Metastatic Castration-resistant Prostate Cancer	Regeneron Pharmaceuticals	Phase 1|Phase 2	129	na
**REGN7075**	1	1	1	CD28	EGFR	NCT04626635	REGN7075 in Combination With Cemiplimab in Adult Participants With Advanced Solid Tumors	Recruiting	Advanced Solid Tumors	Regeneron Pharmaceuticals	Phase 1|Phase 2	312	na
**ABL503**	1	0	3	PDL1	41BB	NCT04762641	This is a Study to Evaluate the Safety and Tolerability of ABL503, and to Determine the Maximum Tolerated Dose (MTD) and Recommended Phase 2 Dose (RP2D) of ABL503 in Subjects With Any Progressive Locally Advanced or Metastatic Solid Tumors	Not yet recruiting	Advanced Solid Tumor	ABL Bio, Inc.	Phase 1	36	na
**ERY974**	1	0	1	CD3	GPC3	NCT02748837	A Study of ERY974 in Patient With Advanced Solid Tumors	Completed	Solid Tumors	Chugai Pharmaceutical	Phase 1	29	ART-Ig

**M701**	1	0	1	CD3	EpCAM	NCT04501744	A Study of M701 (EpCAM and CD3) in Malignant Ascites	Recruiting	Malignant Ascites∣Cancer	Wuhan YZY Biopharma Co., Ltd.	Phase 1	42	na
**M802**	1	0	1	CD3	HER2	NCT04501770	A Study of M802 (HER2 and CD3) in HER2-Positive Advanced Solid Tumors	Recruiting	HER2-Positive Solid Tumors	Wuhan YZY Biopharma Co., Ltd.	Phase 1	32	YBODY
**M1231**	0	0	4	EGFR	MUC1	NCT04695847	M1231 in Participants With Solid Tumors	Recruiting	Metastatic Solid Tumors∣Esophageal Cancer∣Non-Small Cell Lung Cancer	EMD Serono Research & Development Institute, Inc.∣Merck KGaA, Darmstadt, Germany∣EMD Serono	Phase 1	84	na
**ABL001 (NOV1501) (TR009)**	1	0	1	VEGF	DLL4	NCT03292783	This is a Study to Evaluate the Safety and Tolerability of the Study Drug ABL001, and to Determine the Maximum Tolerated Dose and/or Recommended Phase 2 Study Dose of ABL001	Recruiting	Advanced Solid Tumors	ABL Bio, Inc.∣National OncoVenture	Phase 1	39	IgG-scFv
		2				NCT04492033	This is a Study to Evaluate the Safety and Tolerability of the Study Medication ABL001 in Combiation With Irinotecan/Paclitaxel, and to Determine the Maximum Tolerated Dose and/or Recommended Phase 2 Study Dose of ABL001 in Combination With Irinotecan/Paclitaxel.	Recruiting	Advanced Solid Tumors	ABL Bio, Inc.∣National OncoVenture	Phase 1∣Phase 2	18	
**ABL111 (TJ033721)**	na	0	1	Claudin18.2	41BB	NCT04900818	Study of TJ033721 in Subjects With Advanced or Metastatic Solid Tumors	Not yet recruiting	Solid Tumor∣Advanced Cancer∣Metastatic Cancer∣Gastric Cancer∣Gastroesophageal Junction	I-Mab Biopharma Co. Ltd.	Phase 1	108	na

									Carcinoma | Esophageal Adenocarcinoma | Pancreatic Ductal Adenocarcinoma				
**ABL503 (TJ-CD4B)**	0	0	3	PDL1	41BB	NCT04762641	This is a Study to Evaluate the Safety and Tolerability of ABL503, and to Determine the Maximum Tolerated Dose (MTD) and Recommended Phase 2 Dose (RP2D) of ABL503 in Subjects With Any Progressive Locally Advanced or Metastatic Solid Tumors	Recruiting	Advaced Solid Tumors	I-Mab Biopharma Co. Ltd.	Phase 1	39	na
**Ertumaxomab**	1	0	1	CD3	HER2	NCT00452140	Phase II Study With the Trifunctional Antibody Ertumaxomab to Treat Metastatic Breast Cancer Progressing After Endocrine Treatment	Terminated	Metastatic Breast Cancer | Advanced Breast Cancer	Neovii Biotech	Phase 2	40	Triomab
		0				NCT00351858	Safety and Efficacy Study of the Trifunctional Antibody Ertumaxomab to Treat Patients With Advanced or Metastatic Breast Cancer (IV REXBC 02)	Terminated	Breast Cancer	Neovii Biotech	Phase 2	40	
		0				NCT01569412	Open Label, Dose Escalating Study With Ertumaxomab In Patients With HER-2/Neu Expressing Advanced Solid Tumors	Terminated	Her2/Neu Positive Advanced Solid Tumors	Neovii Biotech	Phase 1 | Phase 2	14	
		0				NCT00522457	Phase II Study With the Trifunctional Antibody Ertumaxomab to Treat Metastatic Breast Cancer After	Terminated	Metastatic Breast Cancer | Advanced Breast Cancer	Neovii Biotech | Fresenius Biotech North America	Phase 2	19	

							Progression on Trastuzumab Therapy						
**GS-1423**	1	2	3	CD73	TGFb	NCT03954704	Study of GS-1423 in Participants With Advanced Solid Tumors	Terminated	Advanced Solid Tumors	Gilead Sciences	Phase 1	22	na
**IBI315**	1	0	1	PD1	HER2	NCT04162327	A Phase Ia/Ib Study of IBI315 in Patients With HER2-expressing Advanced Solid Tumor	Recruiting	Advanced Solid Tumor	Innovent Biologics (Suzhou) Co. Ltd.	Phase 1	191	na
**IBI318**	1	2	3	PD1	PDL1	NCT04672928	A Clinical Study to Evaluate the Efficacy and Safety of IBI318 in Combination With Paclitaxel Versus Placebo in Combination With Paclitaxel in Patients With Small Cell Lung Cancer Who Have Failed First-line or Above Chemotherapies	Recruiting	Small Cell Lung Carcinoma	Innovent Biologics (Suzhou) Co. Ltd.	Phase 1	20	na
		0				NCT04611321	Study of IBI318 in Patients With Advanced Cutaneous Squamous Cell Carcinoma	Recruiting	Advanced Cutaneous Squamous Cell Carcinoma	Innovent Biologics (Suzhou) Co. Ltd.	Phase 1|Phase 2	56	
		2				NCT04635527	A Study on the Safety and Effectiveness of IBI318 Combined With Conventional TACE (cTACE) as a Perioperative Treatment for Potentially Resected Hepatocellular Carcinoma	Not yet recruiting	Hepatocellular Carcinoma	Innovent Biologics (Suzhou) Co. Ltd.	Phase 1	20	
		0				NCT03875157	Study of IBI318 in Participants With Advanced Malignancies	Recruiting	Advanced Malignancies	Innovent Biologics (Suzhou) Co. Ltd.	Phase 1	327	
		2				NCT04777084	The Efficacy and Safety of the Bispecific Anti-PD-1/PD-L1 Antibody IBI318 Combined With Lenvatinib in NSCLC.	Recruiting	Non-Small Cell Lung Cancer	Hunan Province Tumor Hospital	Phase 2	30	

**IBI322**	1	0	3	PDL1	CD47	NCT04912466	IBI322 Monotherapy or Combination Therapy in Subjects With Advanced Malignant Tumors.	Not yet recruiting	Advanced Solid Tumor	Innovent Biologics (Suzhou) Co. Ltd.	Phase 1	36	na
		0				NCT04338659	Safety and Efficacy of IBI322 in American Subjects With Advanced Malignant Tumors	Not yet recruiting	Advanced Malignancies	Innovent Biologics (Suzhou) Co. Ltd.	Phase 1	45	
		0				NCT04328831	Safety and Efficacy of IBI322 in Chinese Subjects With Advanced Malignant Tumors	Recruiting	Advanced Malignancies	Innovent Biologics (Suzhou) Co. Ltd.	Phase 1	218	
**CC-1**	1	2	1	CD3	PSMA	NCT04496674	Bispecific PSMAxCD3 Antibody CC-1 in Patients With Squamous Cell Carcinoma of the Lung	Not yet recruiting	Lung Cancer Squamous Cell	German Cancer Research Center | University Hospital Tuebingen	Phase 1 | Phase 2	86	na
		0				NCT04104607	the Bispecific PSMAxCD3 Antibody CC-1 in Patients With Castration Resistant Prostate Carcinoma	Recruiting	Castration-Resistant Prostatic Cancer	University Hospital Tuebingen | German Cancer Research Center	Phase 1	86	
**KN026**	1	0	2	HER2	HER2	NCT04521179	Study of KN026 Combined With KN046 in Patients With Locally Advanced HER2-positive Solid Tumors	Recruiting	HER2-positive Solid Tumors	Jiangsu Alphamab Biopharmaceuticals Co., Ltd | Peking University	Phase 2	30	CRIB
		2				NCT04778982	Study of KN026 in Combination With Palbociclib With or Without Fulvestrant in Patients With Advanced HER2-positive Breast Cancer	Not yet recruiting	HER2-positive Metastatic Breast Cancer	Jiangsu Alphamab Biopharmaceuticals Co., Ltd	Phase 1 | Phase 2	66	
		0				NCT03847168	KN026 in Patients With HER2 Expressing Breast Cancer and Gastric Cancer	Recruiting	Breast Cancer | Gastric/Gastroesophageal Junction Cancer	Jiangsu Alphamab Biopharmaceuticals Co., Ltd	Phase 1	20	
		0				NCT03925974	KN026 in Patients With HER2 Expressing Gastric/Gastroesophageal Junction Cancer	Recruiting	Gastric/Gastroesophageal Junction Cancer	Jiangsu Alphamab Biopharmaceuticals Co., Ltd	Phase 2	50	

		2				NCT04881929	Study of KN026 in Combination With Docetaxel as Neoadjuvant Therapy in HER2-positive Breast Cancer	Not yet recruiting	Breast Cancer	Jiangsu Alphamab Biopharmaceuticals Co., Ltd	Phase 2	30	
		2				NCT04165993	Study of KN026 Monotherapy or Combination Therapy in Patients With Metastatic Breast Cancer	Recruiting	Metastatic Breast Cancer	Jiangsu Alphamab Biopharmaceuticals Co., Ltd	Phase 2	93	
		0				NCT03619681	Trial of KN026 in Patients With HER2-positive Advanced Malignant Breast Cancer and Gastric Cancer	Recruiting	Breast Cancer|Gastric Cancer	Jiangsu Alphamab Biopharmaceuticals Co., Ltd	Phase 1	20	
**KN046**	1	0	3	PDL1	CTLA4	NCT04925947	A Study of KN046 in Patients With Thymic Carcinoma Who Failed Immune Checkpoint Inhibitors	Not yet recruiting	No Results Available	Thymic Carcinoma	Phase 2	29	CRIB
		0				NCT03925870	KN046 in Subjects With Late Stage Esophageal Squamous Cell Carcinoma	Recruiting	No Results Available	Esophageal Squamous Cell Carcinoma	Phase 2	30	
		2				NCT04612712	A Phase 1/2 Study of Donafenib in Combination With KN046 in Advanced Gastrointestinal Tumors	Recruiting	No Results Available	Advanced Gastrointestinal Tumors	Phase 1|Phase 2	42	
		0				NCT03927495	KN046 in Patients With Recurrent or Metastatic Esophageal Squamous Cell Carcinoma	Recruiting	No Results Available	Esophageal Squamous Cell Carcinoma	Phase 2	100	
		0				NCT03872791	A Study of KN046 in Subjects With Locally Advanced or Metastatic Triple-negative Breast Cancer	Recruiting	No Results Available	Triple-negative Breast Cancer	Phase 1|Phase 2	90	
		0				NCT03529526	Study of the Safety, Tolerability, Pharmacokinetics, Immunogenicity and Antitumor Activity of KN046 in	Unknown status	No Results Available	Advanced Solid Tumors	Phase 1	21	

							Subjects With Advanced Solid Tumors						
		0				NCT04474119	KN046 in Subjects With Advanced Squamous Non-small Cell Lung Cancer	Recruiting	No Results Available	Squamous Non-small-cell Lung Cancer	Phase 3	516	
		0				NCT03838848	KN046 in Patients With Advanced Non-small Cell Lung Cancer	Recruiting	No Results Available	Stage IV Non-small Cell Lung Cancer	Phase 2	149	
		0				NCT03733951	KN046 in Subjects With Advanced Solid Tumors and Lymphoma	Recruiting	No Results Available	Advanced Solid Tumors|Lymphoma	Phase 1	285	
		2				NCT04054531	Study of KN046 With Chemotherapy in First Line Advanced NSCLC	Recruiting	No Results Available	Non-small Cell Lung Cancer	Phase 2	50	
		2				NCT04601610	KN046 Combined With Ningatinib in the Treatment of Advanced Hepatocellular Carcinoma	Not yet recruiting	No Results Available	Advanced HCC	Phase 1|Phase 2	70	
		2				NCT04165993	Study of KN026 Monotherapy or Combination Therapy in Patients With Metastatic Breast Cancer	Recruiting	No Results Available	Metastatic Breast Cancer	Phase 2	93	
		2				NCT04542837	The Study of KN046 in Combination With Lenvatinib in Advanced Hepatocellular Carcinoma	Not yet recruiting	No Results Available	HCC	Phase 2	30	
		0				NCT04469725	KN046 (a Humanized PD-L1/CTLA4 Bispecific Single Domain Fc Fusion Protein Antibody) in Subjects With Thymic Carcinoma	Recruiting	Thymic Carcinoma	Jiangsu Alphamab Biopharmaceuticals Co., Ltd	Phase 2	66	
**KN026 and KN046**	1	1	3	PDL1	CTLA4	NCT04521179	Study of KN026 Combined With KN046 in Patients With Locally Advanced HER2-positive Solid Tumors	Recruiting	HER2-positive Solid Tumors	Jiangsu Alphamab Biopharmaceuticals Co., Ltd|Peking University	Phase 2	30	CRIB

		1				NCT04040699	KN026 Combined With KN046 in Subjects With HER2 Positive Solid Tumor	Recruiting	HER2 Positive Solid Tumor	Peking University|Jiangsu Alphamab Biopharmaceuticals Co., Ltd	Phase 1	24	
**Q-1802**	na	0	1	PDL1	Claudin 18.2	NCT04856150	A Study of Q-1802 in Patients With Advanced Solid Tumors	Not yet recruiting	Advanced Solid Tumors	QureBio Ltd.	Phase 1	66	na
**APVO414 (ES414) (MOR209)**	1	0	1	CD3	PSMA	NCT02262910	Study of ES414 in Metastatic Castration-Resistant Prostate Cancer	Completed	Prostate Cancer	Aptevo Therapeutics	Phase 1	35	ADAPTIR
**XmAb18087 (Tidutamab)**	0	0	1	CD3	SSTR2	NCT03411915	A Study of XmAb18087 in Subjects With NET and GIST	Recruiting	Neuroendocrine Tumor|Gastrointestinal Neoplasm	Xencor, Inc.|ICON plc	Phase 1	87	Xmab
		1				NCT04590781	Safety and Efficacy of XmAb18087 ± Pembrolizumab in Advanced Merkel Cell Carcinoma or Extensive-stage Small Cell Lung Cancer	Not yet recruiting	Merkel Cell Carcinoma|Small Cell Lung Cancer	Xencor, Inc.|ICON plc	Phase 1|Phase 2	142	
**XmAb20717 (XmAb717)**	0	0	3	PD1	CTLA4	NCT03517488	A Study of XmAb20717 in Subjects With Selected Advanced Solid Tumors	Recruiting	Melanoma|Breast Carcinoma|Hepatocellular Carcinoma|Urothelial Carcinoma|Squamous Cell Carcinoma of the Head and Neck|Renal Cell Carcinoma|Colorectal Carcinoma|Non-small Cell Lung Carcinoma|Gastric or Gastroesophageal Junction Adenocarcinoma|Endometrial Carcinoma|Mesothelioma|Neuroendocrine	Xencor, Inc.|ICON plc	Phase 1	154	Xmab

									Carcinoma ǀ Cervical Cancer ǀ Small Cell Lung Carcinoma ǀ Squamous Cell Carcinoma of the Anus ǀ Castration-Resistant Prostate Carcinoma ǀ Nasopharyngeal Carcinoma ǀ Cholangiocarcinoma ǀ Basal Cell Carcinoma ǀ Ovarian Carcinoma ǀ Fallopian Tube Carcinoma ǀ Thymoma ǀ Thy mic Carcinoma ǀ Squamous Cell Carcinoma of the Penis ǀ Vulvar Carcinoma ǀ Solid Tumors With Published Evidence of Anti-tumor Activity With Anti-PD1/PDL1 and/or Anti-CTLA4-directed Therapy ǀ Malignant Adnexal Neoplasms ǀ Non -squamous Cell Salivary Gland Carcinoma				
**XmAb22841 (XmAb841)**	0	1	3	CTLA4	LAG3	NCT03849469	A Study of XmAbÂ®22841 Monotherapy & in Combination w/ Pembrolizumab in Subjects w/ Selected	Recruiting	Melanoma ǀ Cervical Carcinoma ǀ Pancreatic Carcinoma ǀ Triple Negative Breast	Xencor, Inc. ǀ ICON Clinical Research	Phase 1	242	Xmab

							Advanced Solid Tumors		Cancer|Hepatocellular Carcinoma|Urothelial Carcinoma|Squamous Cell Carcinoma of the Head and Neck|Nasopharyngeal Carcinoma|Renal Cell Carcinoma|Non-small Cell Lung Carcinoma|Small Cell Lung Carcinoma|Gastric or Gastroesophageal Junction Adenocarcinoma|Advanced or Metastatic Solid Tumors|Prostate Carcinoma|MSI-H|Mismatch Repair Deficiency|Epithelial Ovarian Cancer|Fallopian Tube Cancer|Primary Peritoneal Carcinoma|Intrahepatic Cholangiocarcinoma|Squamous Cell Anal Cancer|Squamous Cell Penile Carcinoma|Squamous Cell Vulvar Carcinoma				
**XmAb23104 (XmAb104)**	0	1	3	PD1	ICOS	NCT03752398	A Study of XmAbÂ®23104 in Subjects With Selected Advanced Solid	Recruiting	Melanoma (Excluding Uveal Melanoma)|Cervical	Xencor, Inc.|ICON plc	Phase 1	234	Xmab

							Tumors (DUET-3)		Carcinoma | Pancreatic Carcinoma | Breast Carcinoma That is Estrogen Receptor, Progesterone Receptor, and Her2 Negative | Hepatocellular Carcinoma | Urothelial Carcinoma | Squamous Cell Carcinoma of the Head and Neck | Nasopharyngeal Carcinoma | Renal Cell Carcinoma | Colorectal Carcinoma | Endometrial Carcinoma | Non-small Cell Lung Carcinoma | Small Cell Lung Cancer | Gastric or Gastroesophageal Junction Adenocarcinoma | Advanced Solid Tumors | Undifferentiated Pleomorphic Sarcoma				
**BCA101**	1	1	3	EGFR	TGFb	NCT04429542	Study of Safety and Tolerability of BCA101 Alone and in Combination With Pembrolizumab in Patients With EGFR-driven Advanced Solid Tumors	Recruiting	TNBC - Triple-Negative Breast Cancer | Head and Neck Squamous Cell Carcinoma | Squamous Cell Carcinoma of Anal Canal | Uveal	Bicara Therapeutics	Phase 1	292	na

									Melanoma ǀ Glioblastoma ǀ Colorectal Cancer ǀ Chordoma ǀ Squamous Cell Carcinoma of the Lung ǀ KRAS G12D ǀ KRAS G13D ǀ EGFR Amplification ǀ Epithelial Ovarian Cancer ǀ Hepatocellular Carcinoma ǀ Anaplastic Thyroid Cancer ǀ Pancreas Cancer				
**EGFR-ErbituxEDV-MIT**	0	2	4	EGFR	EDV-MIT	NCT02687386	A Study of Intravenous EEDVsMit in Children With Recurrent / Refractory Solid or CNS Tumours Expressing EGFR	Recruiting	Solid Tumours ǀ CNS Tumours	Dr David Ziegler ǀ Engeneic Pty Limited ǀ Sydney Children's Hospitals Network	Phase 1	30	na
**EGFR(V)-EDV-Dox**	0	2	4	EGFR	EDV-Dox	NCT02766699	A Study to Evaluate the Safety, Tolerability and Immunogenicity of EGFR(V)-EDV-Dox in Subjects With Recurrent Glioblastoma Multiforme (GBM)	Recruiting	Glioblastoma ǀ Astrocytoma, Grade IV	Engeneic Pty Limited ǀ Johns Hopkins University	Phase 1	20	na
**MM-111**	0	0	1	HER2	HER3	NCT00911898	A Study of MM-111 in Patients With Advanced, Refractory Her2 Amplified, Heregulin Positive Cancers	Completed	Her2 Amplified Solid Tumors ǀ Metastatic Breast Cancer	Merrimack Pharmaceuticals	Phase 1	20	na
		2				NCT01774851	A Study of MM-111 and Paclitaxel With Trastuzumab in Patients HER2 Positive Carcinomas of the Distal Esophagus, Gastroesophageal	Terminated	HER-2 Gene Amplification Esophagus Cancer Gastroesophageal Junction Cancer Stomach Cancer	Merrimack Pharmaceuticals	Phase 2	84	

							(GE) Junction and Stomach						
		2				NCT01097460	MM-111 in Combination With Herceptin in Patients With Advanced Her2 Amplified, Heregulin Positive Breast Cancer	Completed	Breast Neoplasms	Merrimack Pharmaceuticals	Phase 1	16	
		2				NCT01304784	A Study of MM-111 in Combination With Multiple Treatments in Patients With HER2 Positive Cancer	Completed	HER-2 Gene Amplification	Merrimack Pharmaceuticals	Phase 1	100	
**ISB 1302 (GBR 1302)**	1	0	1	CD3	HER2	NCT03983395	Study of ISB 1302 (CD3 Bispecific Ab) in HER2-positive Metastatic Breast Cancer	Terminated	Breast Cancer	Ichnos Sciences SA∣Glenmark Pharmaceuticals S.A.	Phase 1∣Phase 2	1	BEAT
		0				NCT02829372	Phase 1 Study of Single Agent GBR 1302 in Subjects With HER2 Positive Cancers	Terminated	HER2 Expressing Solid Tumours	Ichnos Sciences SA∣Glenmark Pharmaceuticals S.A.	Phase 1	36	
**MBS301**	1	0	2	HER2	HER2	NCT03842085	Phase I Clinical Study of MBS301 in Treatment of HER2 Positive Recurrent or Metastatic Malignant Solid Tumor	Unknown status	HER2-positive Recurrent or Metastatic Malignant Solid Tumor	Beijing Mabworks Biotech Co., Ltd.	Phase 1	34	KIH
**JNJ-61186372 (Amivantamab)**	1	2	2	EGFR	cMET	NCT02609776	Study of Amivantamab, a Human Bispecific EGFR and cMet Antibody, in Participants With Advanced Non-Small Cell Lung Cancer	Recruiting	Non-Small-Cell Lung Cancer	Janssen Research & Development, LLC	Phase 1	460	DuoBody
		0				NCT04606381	A Study of Amivantamab Subcutaneous (SC) Administration for the Treatment of Advanced Solid Malignancies	Recruiting	Advanced Solid Malignancies	Janssen Research & Development, LLC	Phase 1	80	
		0				NCT04945733	A Study of Amivantamab in Participants With Previously Treated Advanced or	Not yet recruiting	Stomach Neoplasms∣Esophageal Neoplasms	Janssen Pharmaceutical K.K.	Phase 2	60	

							Metastatic Gastric or Esophageal Cancer						
		2				NCT04538664	A Study of Combination Amivantamab and Carboplatin-Pemetrexed Therapy, Compared With Carboplatin-Pemetrexed, in Participants With Advanced or Metastatic Non-Small Cell Lung Cancer Characterized by Epidermal Growth Factor Receptor (EGFR) Exon 20 Insertions	Recruiting	Carcinoma, Non-Small-Cell Lung	Janssen Research & Development, LLC	Phase 3	300	
		2				NCT04077463	A Study of Lazertinib as Monotherapy or in Combination With Amivantamab in Participants With Advanced Non-small Cell Lung Cancer	Recruiting	Carcinoma, Non-Small-Cell Lung	Janssen Research & Development, LLC	Phase 1	520	
		2				NCT04487080	A Study of Amivantamab and Lazertinib Combination Therapy Versus Osimertinib in Locally Advanced or Metastatic Non-Small Cell Lung Cancer	Recruiting	Carcinoma, Non-Small-Cell Lung	Janssen Research & Development, LLC	Phase 3	1000	
**JNJ-63898081**	1	0	1	CD3	PSMA	NCT03926013	A Study of JNJ-63898081 in Participants With Advanced Stage Solid Tumors	Recruiting	metastatic castration-resistant prostate cancer (mCRPC), or renal cell carcinoma (RCC)	Janssen Research & Development, LLC	Phase 1	90	na
**JNJ-70218902**	1	0	na	na	na	NCT04397276	A Study of JNJ-70218902 in Participants With Advanced Stage Solid Tumors	Recruiting	Metastatic Castration-resistant Prostate Cancer	Janssen Research & Development, LLC	Phase 1	70	na
**Navicixizumab (OMP-305B83)**	1	2	1	VEGF	DLL4	NCT03035253	A Study of OMP-305B83 in Subjects With Metastatic Colorectal Cancer	Terminated	Metastatic Colorectal Cancer	OncoMed Pharmaceuticals, Inc.|Mereo BioPharma	Phase 1	16	na

		2				NCT03030287	A Phase 1b Study of OMP-305B83 Plus Paclitaxel in Subjects With Ovarian, Peritoneal or Fallopian Tube Cancer	Completed	Cancer Ovaries丨Cancer Peritoneal丨Cancer, Fallopian Tube	OncoMed Pharmaceuticals, Inc.丨Mereo BioPharma	Phase 1	44	
		0				NCT02298387	A Phase 1 Study of OMP-305B83 in Subjects With Solid Tumors	Completed	Advanced Solid Tumor Malignancies	OncoMed Pharmaceuticals, Inc.丨Mereo BioPharma	Phase 1	71	
**MGD007**	1	0	1	CD3	gpA33	NCT02248805	Phase 1 Study of MGD007 in Relapsed/Refractory Metastatic Colorectal Carcinoma	Completed	Colorectal Carcinoma	MacroGenics	Phase 1	95	Fc-DART
		0				NCT03531632	MGD007 Combined With MGA012 in Relapsed/Refractory Metastatic Colorectal Cancer	Active, not recruiting	Colorectal Cancer Metastatic	MacroGenics	Phase 1丨Phase 2	52	
**MGD009**	1	1	1	CD3	B7H3	NCT03406949	MGD009/MGA012 Combination in Relapsed/Refractory Cancer	Active, not recruiting	Advaced Solid Tumors	MacroGenics	Phase 1	25	Fc-DART
		0				NCT02628535	Safety Study of MGD009 in B7-H3-expressing Tumors	Terminated	B7-H3-expressing tumors	MacroGenics	Phase 1	67	
**MGD013 (Tebotelimab)**	1	2	3	PD1	LAG3	NCT04082364	Combination Margetuximab, INCMGA00012, MGD013, and Chemotherapy Phase 2/3 Trial in HER2+ Gastric/GEJ Cancer (MAHOGANY)	Recruiting	Gastric Cancer丨Gastroesophageal Junction Cancer丨HER2-positive Gastric Cancer	MacroGenics丨Zai Lab (Shanghai) Co., Ltd.	Phase 2丨Phase 3	860	Fc-DART
		2				NCT03219268	A Study of MGD013 in Patients With Unresectable or Metastatic Neoplasms	Recruiting	Advanced Solid Tumors丨Hematologic Neoplasms丨Ovarian Cancer丨HER2-positive Breast Cancer丨Non Small Cell Lung Cancer丨Small-cell Lung Cancer丨Squamous Cell Carcinoma	MacroGenics	Phase 1	352	

									of Head and Neck∣Cholangiocarcinoma∣Cervical Cancer∣TNBC - Triple-Negative Breast Cancer				
		0				NCT04653038	A Study to Evaluate the Safety and Efficacy of MGD013 in Patients With Melanoma	Recruiting	Unresectable, Recurrent or Metastatic Melanoma∣Untreated Mucosal or Acral Lentiginous Melanoma	Zai Lab (Shanghai) Co., Ltd.	Phase 1	160	
		2				NCT04212221	MGD013 Monotherapy and Combination With Brivanib Dose Escalation and Expansion Study in Advanced Liver Cancer Patients	Recruiting	Advanced Hepatocellular Carcinoma (HCC)	Zai Lab (Shanghai) Co., Ltd.	Phase 1∣Phase 2	300	
		2				NCT04178460	A Study of Niraparib Combined With MGD013 in Patients With Advanced or Metastatic Solid Tumor Who Failed Prior Treatment	Recruiting	Gastric Cancer∣Triple Negative Breast Cancer∣Biliary Tract Carcinoma∣Endometrial Carcinoma	Zai Lab (Shanghai) Co., Ltd.	Phase 1	164	
		1				NCT04129320	Enoblituzumab Plus MGA012 or MGD013 in Squamous Cell Carcinoma of the Head and Neck	Withdrawn	Head and Neck Cancer∣Squamous Cell Carcinoma of Head and Neck	MacroGenics	Phase 2∣Phase 3	0	
		1				NCT04634825	Enoblituzumab Plus Retifanlimab or Tebotelimab in Head and Neck Cancer	Recruiting	Head and Neck Cancer∣Head and Neck Neoplasms∣Head and Neck Squamous Cell Carcinoma	MacroGenics	Phase 2	80	
**MGD019**	1	0	3	PD1	CTLA4	NCT03761017	MGD019 DART Protein in Unresectable/Metastatic Cancer	Recruiting	Solid Tumor, AdultAdvanced CancerSquamous Cell Non Small Cell Lung CancerColorectal	MacroGenics	Phase 1	287	Fc-DART

									Cancer-Prostate Cancer MetastaticCutaneous Melanoma				
**AMG 110 (MT110) (Solitomab)**	0	0	1	CD3	EpCAM	NCT00635596	Phase I Study of MT110 in Lung Cancer (Adenocarcinoma and Small Cell), Gastric Cancer or Adenocarcinoma of the Gastro-Esophageal Junction, Colorectal Cancer, Breast Cancer, Hormone-Refractory Prostate Cancer, and Ovarian Cancer (MT110-101)	Completed	Solid Tumors	Amgen	Phase 1	65	BITE
**AMG 160**	1	1	1	CD3	PSMA	NCT03792841	Safety, Tolerability, Pharmacokinetics, and Efficacy of AMG 160 in Subjects With mCRPC	Recruiting	Metastatic Castration-resistant Prostate Cancer	Amgen	Phase 1	288	HLE-BITE
		3				NCT04631601	Safety and Efficacy of Therapies for Metastatic Castration-resistant Prostate Cancer (mCRPC)	Recruiting	Metastatic Castration-resistant Prostate Cancer	Amgen	Phase 1	105	
		0				NCT04822298	Study of AMG 160 in Subjects With Non-Small Cell Lung Cancer	Not yet recruiting	Non-small Cell Lung Cancer丨NSCLC	Amgen	Phase 1	50	
**AMG 211 (MEDI-565)**	0	0	1	CD3	CEA	NCT01284231	A Study to Evaluate the Safety and Tolerability of MEDI-565 in Adults With Gastrointestinal Adenocarcinomas	Completed	Gastrointestinal Adenocarcinomas	MedImmune LLC	Phase 1	78	BITE
		0				NCT02291614	A Phase 1 Study of AMG 211 in Participants With Advanced Gastrointestinal Cancer	Terminated	Gastrointestinal Adenocarcinomas	MedImmune LLC	Phase 1	45	
**AMG 509**	0	0	1	CD3	STEAP-1	NCT04221542	A Phase 1 Study Evaluating the Safety, Tolerability,	Recruiting	Prostate Cancer	Amgen	Phase 1	110	

							Pharmacokinetics, and Efficacy of AMG 509 in Subjects With Metastatic Castration-Resistant Prostate Cancer						
**AMG 596**	0	1	1	CD3	EGFRvIII	NCT03296696	Phase 1/1b Study to Evaluate Safety, Tolerability, Pharmacokinetics and Pharmacodynamics of AMG 596 as Monotherapy and in Combination With AMG 404 in Subjects With Glioblastoma or Malignant Glioma Expressing Mutant Epidermal Growth Factor Receptor Variant III (EGFRvIII)	Active, not recruiting	Glioblastoma	Amgen	Phase 1	30	BITE
**AMG 757**	1	1	1	CD3	DLL3	NCT04885998	AMG 757 and AMG 404 in Subjects With Small Cell Lung Cancer (SCLC)	Not yet recruiting	Small Cell Lung Carcinoma	Amgen	Phase 1	40	HLE-BITE
		1				NCT03319940	Study Evaluating Safety, Tolerability and PK of AMG 757 in Adults With Small Cell Lung Cancer	Recruiting	Small Cell Lung Carcinoma	Amgen	Phase 1	332	
		0				NCT04702737	A Study of AMG 757 in Participants With Neuroendocrine Prostate Cancer	Not yet recruiting	Neuroendocrine Prostate Cancer	Amgen	Phase 1	60	
**AMG 910**	1	0	1	CD3	Claudin 18.2	NCT04260191	Study of AMG 910 in Subjects With CLDN18.2-Positive Gastric and Gastroesophageal Junction Adenocarcinoma	Recruiting	Gastric and Gastroesophageal Junction Adenocarcinoma	Amgen	Phase 1	70	na
**Dilpacimab (ABT-165)**	1	2	1	VEGF	DLL4	NCT03368859	A Study of ABT-165 Plus FOLFIRI vs Bevacizumab Plus FOLFIRI in Subjects With Metastatic	Terminated	Metastatic Colorectal Cancer	AbbVie	Phase 2	70	DVD-Ig

							Colorectal Cancer Previously Treated With Fluoropyrimidine, Oxaliplatin and Bevacizumab						
		2				NCT01946074	A Study of ABT-165 in Subjects With Solid Tumors	Active, not recruiting	Advanced Solid Tumors	AbbVie	Phase 1	100	
**IMC-C103C**	0	1	1	CD3	MAGE-A4	NCT03973333	Safety and Efficacy of IMC-C103C as Monotherapy and in Combination With Atezolizumab	Recruiting	Select Advanced Solid Tumors	Immunocore Ltd	Phase 1|Phase 2	144	na
**IMC-F106C**	0	1	1	CD3	PRAME	NCT04262466	Safety and Efficacy of IMC-F106C as a Single Agent and in Combination With Checkpoint Inhibitors	Recruiting	Select Advanced Solid Tumors	Immunocore Ltd	Phase 1|Phase 2	170	na
**M7824 (Bintrafusp alfa)**	1	0	3	PDL1	TGFb	NCT02517398	MSB0011359C (M7824) in Metastatic or Locally Advanced Solid Tumors	Active, not recruiting	Solid Tumors	EMD Serono Research & Development Institute, Inc.|Merck KGaA, Darmstadt, Germany|EMD Serono	Phase 1	600	na
		0				NCT02699515	MSB0011359C (M7824) in Subjects With Metastatic or Locally Advanced Solid Tumors	Active, not recruiting	Solid Tumors	Merck KGaA, Darmstadt, Germany	Phase 1	114	
		3				NCT02723955	Dose Escalation and Expansion Study of GSK3359609 in Participants With Selected Advanced Solid Tumors (INDUCE-1)	Recruiting	Neoplasms	GlaxoSmithKline|Merck Sharp & Dohme Corp.	Phase 1	873	
		0				NCT03427411	M7824 in Subjects With HPV Associated Malignancies	Active, not recruiting	Human Papilloma Virus|Cervical Cancer|Oropharyngeal Cancer|Anal Cancer|Vaginal or Penile Cancer	National Cancer Institute (NCI)|National Institutes of Health Clinical Center (CC)	Phase 2	57	
		0				NCT03436563	M7824 in Patients With Metastatic Colorectal Cancer or With Advanced	Recruiting	Colon Adenocarcinoma|High-Frequency	M.D. Anderson Cancer Center|National Cancer Institute (NCI)	Phase 1|Phase 2	74	

		Solid Tumors With Microsatellite Instability		Microsatellite Instability | Metastatic Malignant Solid Neoplasm | Rectal Adenocarcinoma | Refractory Colorectal Carcinoma | Stage IV Colon Cancer AJCC v8 | Stage IV Rectal Cancer AJCC v8 | Stage IVA Colon Cancer AJCC v8 | Stage IVA Rectal Cancer AJCC v8 | Stage IVB Colon Cancer AJCC v8 | Stage IVB Rectal Cancer AJCC v8			
2	NCT03451773	M7824 (MSB0011359C) in Combination With Gemcitabine in Adults With Previously Treated Advanced Adenocarcinoma of the Pancreas	Completed	Cancer of Pancreas | Pancreas Cancer | Pancreatic Adenocarcinoma | Pancreatic Cancer | Pancreatic Neoplasms	National Cancer Institute (NCI) | National Institutes of Health Clinical Center (CC)	Phase 1 | Phase 2	7
2	NCT03524170	Radiation Therapy and M7824 in Treating Patients With Metastatic Hormone Receptor Positive, HER2 Negative Breast Cancer	Recruiting	Anatomic Stage IV Breast Cancer AJCC v8 | Metastatic Breast Carcinoma | Prognostic Stage IV Breast Cancer AJCC v8	M.D. Anderson Cancer Center | National Cancer Institute (NCI)	Phase 1	20
2	NCT03554473	M7824 and Topotecan or Temozolomide in Relapsed Small Cell Lung Cancers	Recruiting	Carcinoma, Small Cell | Lung Cancer | Small Cell Lung Cancer	National Cancer Institute (NCI) | National Institutes of Health Clinical Center (CC)	Phase 1 | Phase 2	67
2	NCT03579472	M7824 and Eribulin Mesylate in Treating	Recruiting	Anatomic Stage IV Breast	M.D. Anderson Cancer Center | National	Phase 1	20

		Patients With Metastatic Triple Negative Breast Cancer		Cancer AJCC v8 ǀ Metastatic Triple-Negative Breast Carcinoma ǀ Prognostic Stage IV Breast Cancer AJCC v8	Cancer Institute (NCI)		
0	NCT03620201	M7824 in Treating Patients With Stage II-III HER2 Positive Breast Cancer	Recruiting	Anatomic Stage II Breast Cancer AJCC v8 ǀ Anatomic Stage IIA Breast Cancer AJCC v8 ǀ Anatomic Stage IIB Breast Cancer AJCC v8 ǀ Anatomic Stage III Breast Cancer AJCC v8 ǀ Anatomic Stage IIIA Breast Cancer AJCC v8 ǀ Anatomic Stage IIIB Breast Cancer AJCC v8 ǀ Anatomic Stage IIIC Breast Cancer AJCC v8 ǀ Prognostic Stage II Breast Cancer AJCC v8 ǀ Prognostic Stage IIA Breast Cancer AJCC v8 ǀ Prognostic Stage IIB Breast Cancer AJCC v8 ǀ Prognostic Stage III Breast Cancer AJCC v8 ǀ Prognostic Stage IIIA Breast Cancer AJCC v8 ǀ Prognostic Stage IIIB Breast Cancer AJCC	M.D. Anderson Cancer Center ǀ National Cancer Institute (NCI)	Phase 1	20

				v8 | Prognostic Stage IIIC Breast Cancer AJCC v8			
0	NCT03833661	M7824 Monotherapy in Locally Advanced or Metastatic Second Line (2L) Biliary Tract Cancer (Cholangiocarcinoma and Gallbladder Cancer)	Active, not recruiting	Biliary Tract Cancer | Cholangiocarcinoma | Gallbladder Cancer	EMD Serono Research & Development Institute, Inc. | Merck KGaA, Darmstadt, Germany | EMD Serono	Phase 2	159
2	NCT03840915	M7824 in Combination With Chemotherapy in Stage IV Non-small Cell Lung Cancer (NSCLC)	Active, not recruiting	Carcinoma, Non-Small-Cell Lung	EMD Serono Research & Development Institute, Inc. | Merck KGaA, Darmstadt, Germany | EMD Serono	Phase 1 | Phase 2	70
2	NCT04066491	Gemcitabine Plus Cisplatin With or Without Bintrafusp Alfa (M7824) in Participants With 1L Biliary Tract Cancer (BTC)	Recruiting	Biliary Tract Cancer | Cholangiocarcinoma | Gallbladder Cancer	EMD Serono Research & Development Institute, Inc. | Merck KGaA, Darmstadt, Germany | EMD Serono	Phase 2 | Phase 3	512
2	NCT04220775	Bintrafusp Alfa and Stereotactic Body Radiation Therapy for the Treatment of Recurrent or Second Primary Head and Neck Squamous Cell Cancer	Recruiting	Recurrent Head and Neck Squamous Cell Carcinoma | Second Primary Squamous Cell Carcinoma of the Head and Neck	M.D. Anderson Cancer Center | National Cancer Institute (NCI)	Phase 1 | Phase 2	21
2	NCT04235777	Bintrafusp Alfa (M7824) and NHS-IL12 (M9241) Alone and in Combination With Stereotactic Body Radiation Therapy (SBRT) in Adults With Metastatic Non-Prostate Genitourinary Malignancies	Recruiting	Urothelial Cancer | Bladder Cancer | Genitourinary Cancer | Urogenital Neoplasms | Urogenital Cancer	National Cancer Institute (NCI) | National Institutes of Health Clinical Center (CC)	Phase 1	66
1	NCT04247282	Anti-PD-L1/TGF-beta Trap (M7824) Alone and in	Recruiting	Head and Neck Cancer | Head	National Cancer Institute (NCI) | National Institutes of	Phase 1 | Phase 2	40

		Combination With TriAd Vaccine and N-803 for Resectable Head and Neck Squamous Cell Carcinoma Not Associated With Human Papillomavirus Infection		and Neck Neoplasms	Health Clinical Center (CC)		
1	NCT04287868	Combination Immunotherapy in Subjects With Advanced HPV Associated Malignancies	Recruiting	Cervical Cancer|HPV Cancers|Anal Cancer|Oropharyngeal Cancer|Vulvar, Vaginal, Penile, Rectal Cancer	National Cancer Institute (NCI)|National Institutes of Health Clinical Center (CC)	Phase 1|Phase 2	56
3	NCT04296942	BN-Brachyury, Entinostat, Ado-trastuzumab Emtansine and M7824 in Advanced Stage Breast Cancer (BrEAsT)	Recruiting	Breast Cancer|Triple Negative Breast Cancer|HER2+ Breast Cancer|Hormone Receptor Negative Breast Cancer|Metastatic Breast Cancer	National Cancer Institute (NCI)|National Institutes of Health Clinical Center (CC)	Phase 1	65
1	NCT04303117	NHS-IL12 Monotherapy and in Combination With M7824 in Advanced Kaposi Sarcoma	Recruiting	Kaposi Sarcoma	National Cancer Institute (NCI)|National Institutes of Health Clinical Center (CC)	Phase 1|Phase 2	56
0	NCT04349280	A Study to Evaluate the Efficacy and Safety of Bintrafusp Alfa (M7824) Monotherapy in Metastatic or Locally Advanced Urothelial Cancer	Recruiting	Neoplasms	GlaxoSmithKline|Merck KGaA, Darmstadt, Germany	Phase 1	40
2	NCT04396535	Docetaxel With or Without Bintrafusp Alfa for the Treatment of Advanced Non-small Cell Lung Cancer	Recruiting	Advanced Lung Non-Small Cell Carcinoma|Stage III Lung Cancer AJCC v8|Stage IIIA Lung Cancer AJCC v8|Stage IIIB Lung Cancer	Mayo Clinic|National Cancer Institute (NCI)	Phase 2	80

				AJCC v8ǀStage IIIC Lung Cancer AJCC v8ǀStage IV Lung Cancer AJCC v8ǀStage IVA Lung Cancer AJCC v8ǀStage IVB Lung Cancer AJCC v8			
0	NCT04396886	Bintrafusp Alfa in Previously Treated Patients With Recurrent and Metastatic (R/M) Non-keratinizing Nasopharyngeal Carcinoma (NPC)	Recruiting	Nasopharyngeal CarcinomaǀRecurrent CarcinomaǀMetastatic CancerǀNon-keratinizing Carinoma	The University of Hong KongǀMerck Healthcare KGaA, Darmstadt, Germany, an affiliate of Merck KGaA, Darmstadt, Germany	Phase 2	37
0	NCT04428047	Evaluation of Bintrafusp Alfa in Operable and Untreated Head and Neck Squamous Cell Carcinoma	Recruiting	Squamous Cell Carcinoma of Head and Neck	UNICANCERǀMerck KGaA, Darmstadt, Germany	Phase 2	59
1	NCT04432597	HPV Vaccine PRGN-2009 Alone or in Combination With Anti-PDL1/TGF-Beta Trap (M7824) in Subjects With HPV Associated Cancers	Recruiting	HPV Positive CancerǀVulvar, Vaginal, Penile, Rectal CancerǀAnal CancerǀOropharyngeal CancerǀCervical Cancer	National Cancer Institute (NCI)ǀNational Institutes of Health Clinical Center (CC)	Phase 1ǀPhase 2	76
2	NCT04481256	TGF-b And PDL-1 Inhibition in Esophageal Squamous Cell Carcinoma Combined With Chemoradiation Therapy	Recruiting	Carcinoma, Squamous CellǀOesophageal Cancer	Academisch Medisch Centrum - Universiteit van Amsterdam (AMC-UvA)ǀUMC UtrechtǀCatharina Ziekenhuis EindhovenǀVerbeeten Insituut TilburgǀElisabeth-TweeSteden ZiekenhuisǀLeiden University Medical CenterǀRadiotherapeutic Institute FrieslandǀMedisch Centrum	Not Applicable	49

					Leeuwarden|Radiotherapy Group Deventer|Deventer Ziekenhuis|Rijnstate Hospital|Erasmus Medical Center|The Netherlands Cancer Institute|Maastro Clinic, The Netherlands|Zuyderland Medisch Centrum|Isala|University Medical Center Groningen|ZGT		
0	NCT04489940	Bintrafusp Alfa in High Mobility Group AT-Hook 2 (HMGA2) Expressing Triple Negative Breast Cancer	Recruiting	Triple Negative Breast Neoplasms	EMD Serono Research & Development Institute, Inc.|Merck KGaA, Darmstadt, Germany|EMD Serono	Phase 2	29
1	NCT04491955	Phase II Trial of Combination Immunotherapy in Subjects With Advanced Small Bowel and Colorectal Cancers	Recruiting	Small Bowel Cancers|Colorectal Cancers	National Cancer Institute (NCI)|National Institutes of Health Clinical Center (CC)	Phase 2	80
0	NCT04501094	A Phase II Study of Bintrafusp Alfa (M7824) in Checkpoint Inhibitor Naive and Refractory Subjects With Urothelial Carcinoma	Recruiting	Urothelial Cancer	National Cancer Institute (NCI)|National Institutes of Health Clinical Center (CC)	Phase 2	75
2	NCT04551950	Bintrafusp Alfa Combination Therapy in Participants With Cervical Cancer (INTR@PID 046)	Recruiting	Cervical Cancer	EMD Serono Research & Development Institute, Inc.|Merck KGaA, Darmstadt, Germany|EMD Serono	Phase 1	25
1	NCT04574583	Phase I/II Trial Investigating the Safety, Tolerability, Pharmacokinetics, Immune and	Recruiting	Metastatic Cancer|Solid Tumors	National Cancer Institute (NCI)|National Institutes of Health Clinical Center (CC)	Phase 1|Phase 2	105

		Clinical Activity of SX-682 in Combination With BinTrafusp Alfa (M7824 or TGF-beta "Trap"/PD-L1) With CV301 TRICOM in Advanced Solid Tumors (STAT)					
2	NCT04633252	Bintrafusp Alfa (M7824) and M9241 in Combination With Docetaxel in Adults With Metastatic Castration Sensitive and Castration Resistant Prostate Cancer	Recruiting	Cancer Of Prostate|Prostate Neoplasms	National Cancer Institute (NCI)|National Institutes of Health Clinical Center (CC)	Phase 1|Phase 2	86
2	NCT04648826	Aerosolized Azacytidine as Epigenetic Priming for Bintrafusp Alfa-Mediated Immune Checkpoint Blockade in Patients With Unresectable Pulmonary Metastases From Sarcomas, Germ Cell Tumors, or Epithelial Malignancies	Not yet recruiting	Sarcomas|Melanomas|Germ Cell Tumors|Epithelial Malignancies (Excluding Lung and Renal Cell Carcinomas)|Pulmonary Metastases	National Cancer Institute (NCI)|National Institutes of Health Clinical Center (CC)	Phase 1|Phase 2	42
2	NCT04708067	Hypofractionated Radiation Therapy and Bintrafusp Alfa for the Treatment of Advanced Intrahepatic Cholangiocarcinoma	Not yet recruiting	Locally Advanced Intrahepatic Cholangiocarcinoma|Metastatic Intrahepatic Cholangiocarcinoma|Stage III Intrahepatic Cholangiocarcinoma AJCC v8|Stage IIIA Intrahepatic Cholangiocarcinoma AJCC v8|Stage IIIB Intrahepatic Cholangiocarcinoma	M.D. Anderson Cancer Center	Phase 1	15

				AJCC v8 | Stage IV Intrahepatic Cholangiocarcinoma AJCC v8			
1	NCT04708470	Phase I/II Trial of the Combination of Bintrafusp Alfa (M7824), Entinostat and NHS-IL12 (M9241) in Patients With Advanced Cancer	Not yet recruiting	Cancer | Solid Tumor | Metastatic Checkpoint Refractory HPV Associated Malignancies | Microsatellite Stable Colon Cancer (MSS)	National Cancer Institute (NCI) | National Institutes of Health Clinical Center (CC)	Phase 1 | Phase 2	60
0	NCT04727541	Neoadjuvant Bintrafusp Alfa in Patients With Resectable Biliary Tract Cancer	Not yet recruiting	Biliary Tract Cancer | Cholangiocarcinoma	AIO-Studien-gGmbH | Merck Serono GmbH, Germany	Phase 2	24
3	NCT04756505	Immunotherapy (NHS-IL12 & Bintrafusp Alfa) and Radiation Therapy for the Treatment of Hormone Receptor Positive, HER2 Negative Metastatic Breast Cancer, the REINA Trial	Not yet recruiting	Anatomic Stage IV Breast Cancer AJCC v8 | Hormone Receptor Positive Breast Adenocarcinoma | Metastatic Breast Carcinoma | Metastatic HER2 Negative Breast Adenocarcinoma | Prognostic Stage IV Breast Cancer AJCC v8	M.D. Anderson Cancer Center	Phase 1	20
2	NCT04789668	Bintrafusp Alfa and Pimasertib for the Treatment of Patients With Brain Metastases	Recruiting	Anatomic Stage IV Breast Cancer AJCC v8 | Clinical Stage IV Cutaneous Melanoma AJCC v8 | Hematopoietic and Lymphoid Cell Neoplasm | Hormone Receptor Positive Breast	M.D. Anderson Cancer Center	Phase 1 | Phase 2	36

				Adenocarcinoma|Metastatic Lung Non-Small Cell Carcinoma|Metastatic Malignant Neoplasm in the Brain|Metastatic Malignant Solid Neoplasm|Metastatic Melanoma|Metastatic Triple-Negative Breast Carcinoma|Pathologic Stage IV Cutaneous Melanoma AJCC v8|Prognostic Stage IV Breast Cancer AJCC v8|Stage IV Lung Cancer AJCC v8|Stage IVA Lung Cancer AJCC v8|Stage IVB Lung Cancer AJCC v8			
2	NCT04835896	Study of M7824 and Paclitaxel Combination as a Second-line Treatment in Patients With Recurrent/Metastatic Gastric Cancer	Not yet recruiting	Recurrent/Metastatic Gastric Cancer	Yonsei University	Phase 1|Phase 2	49
0	NCT03707587	M7824 in People With Recurrent Respiratory Papillomatosis	Active, not recruiting	Recurrent Respiratory Papillomatosis|Respiratory Papillomatosis|Laryngeal Papilloma, Recurrent|Human Papilloma Virus	National Cancer Institute (NCI)|National Institutes of Health Clinical Center (CC)	Phase 2	9
0	NCT04417660	Bintrafusp Alfa (M7824) in Subjects With Thymoma and Thymic Carcinoma	Recruiting	Thymic Epithelial Tumor|Recurrent Thymoma|Thymic Cancer	National Cancer Institute (NCI)|National Institutes of Health Clinical Center (CC)	Phase 2	38

3	NCT04327986	Immune Checkpoint Inhibitor M7824 and the Immunocytokine M9241 in Combination With Stereotactic Body Radiation Therapy (SBRT) in Adults With Advanced Pancreas Cancer	Recruiting	Histologically or Cytologically Confirmed Pancreatic Cancer|Unresectable or Borderline Resectable Pancreatic Cancer|Pancreatic Neoplasms|Pancreatic Cancer|Metastatic Pancreatic Cancer	National Cancer Institute (NCI)|National Institutes of Health Clinical Center (CC)	Phase 1|Phase 2	52
0	NCT03631706	M7824 Versus Pembrolizumab as a First-line (1L) Treatment in Participants With Programmed Death-ligand 1 (PD-L1) Expressing Advanced Non-small Cell Lung Cancer (NSCLC)	Active, not recruiting	Non-small Cell Lung Cancer	EMD Serono Research & Development Institute, Inc.|Merck KGaA, Darmstadt, Germany|EMD Serono	Phase 3	584
3	NCT03840902	M7824 With cCRT in Unresectable Stage III Non-small Cell Lung Cancer (NSCLC)	Recruiting	Non-small Cell Lung Cancer	EMD Serono Research & Development Institute, Inc.|Merck KGaA, Darmstadt, Germany|EMD Serono	Phase 2	350
3	NCT03493945	Phase I/II Study of Immunotherapy Combination BN-Brachyury Vaccine, M7824, N-803 and Epacadostat (QuEST1)	Recruiting	Metastatic Prostate Cancer|Prostate Cancer|Prostate Neoplasm|Advanced Solid Tumors|Solid Tumor	National Cancer Institute (NCI)|National Institutes of Health Clinical Center (CC)	Phase 1|Phase 2	113
0	NCT04246489	Bintrafusp Alfa Monotherapy in Platinum-Experienced Cervical Cancer	Active, not recruiting	Uterine Cervical Neoplasms	EMD Serono Research & Development Institute, Inc.|Merck KGaA, Darmstadt, Germany|EMD Serono	Phase 2	146

		0				NCT04595149	Tapestry: Addition of TGFb and PDL-1 Inhibition to Definitive Chemoradiation in Esophageal Squamous Cell Carcinoma	Recruiting	Neoplasm, Esophagus∣Malignant Esophagus Tumor∣Neoplasm, Esophageal	Academisch Medisch Centrum - Universiteit van Amsterdam (AMC-UvA)	Phase 2	52	
		0				NCT04560686	Bintrafusp Alfa Before Surgery for the Treatment of Untreated Resectable Non-small Cell Lung Cancer	Recruiting	Resectable Lung Non-Small Cell Carcinoma∣Stage I Lung Cancer AJCC v8∣Stage IA1 Lung Cancer AJCC v8∣Stage IA2 Lung Cancer AJCC v8∣Stage IA3 Lung Cancer AJCC v8∣Stage IB Lung Cancer AJCC v8∣Stage II Lung Cancer AJCC v8∣Stage IIA Lung Cancer AJCC v8∣Stage IIB Lung Cancer AJCC v8∣Stage IIIA Lung Cancer AJCC v8∣Stage IIIB Lung Cancer AJCC v8	M.D. Anderson Cancer Center∣National Cancer Institute (NCI)	Phase 2	23	
		1				NCT03315871	Combination Immunotherapy in Biochemically Recurrent Prostate Cancer	Recruiting	Prostate Cancer	National Cancer Institute (NCI)∣National Institutes of Health Clinical Center (CC)	Phase 2	34	
**BAY2010112 (Pasotuxizumab)**	0	0	1	CD3	PSMA	NCT01723475	First-in-man Dose Escalation Study of BAY2010112 in Patients With Prostate Cancer	Completed	Prostatic Neoplasms	Bayer	Phase 1	47	BITE
**HPN-424**	0	0	1	CD3	PSMA	NCT03577028	Study of HPN424 in Patients With Advanced Prostate Cancer	Recruiting	Advanced Prostate Cancer	Harpoon Therapeutics	Phase 1∣Phase 2	40	DART
**BTRC4017A (RG6194)**	1	0	1	CD3	HER2	NCT03448042	A Phase I Study of BTRC4017A in Participants	Recruiting	Solid Tumors	Genentech, Inc.	Phase 1	449	na

							With Locally Advanced or Metastatic HER2-Expressing Cancers						
**MEDI5752**	1	1, 2	3	PD1	CTLA4	NCT03819465	A Study of Novel Anti-cancer Agents in Patients With Previously Untreated NSCLC (MAGELLAN)	Active, not recruiting	Metastatic Non-Small Cell Lung Cancer (NSCLC)	MedImmune LLC	Phase 1	212	DuetMab/KIH
		1, 2				NCT03530397	A Study to Evaluate MEDI5752 in Subjects With Advanced Solid Tumors	Recruiting	Selected Advanced Solid Tumors	MedImmune LLC	Phase 1	261	
		2				NCT04522323	A Study to Evaluate MEDI5752 and Axitinib in Subjects With Advanced Renal Cell Carcinoma	Active, not recruiting	Advanced Renal Cell Carcinoma	MedImmune LLC	Phase 1	77	
**PRS-343 (Cinrebafusp Alfa)**	0	1	1	HER2	41BB	NCT03650348	PRS-343 in Combination With Atezolizumab in HER2-Positive Solid Tumors	Active, not recruiting	HER2-positive Solid Tumor (Breast, Gastric, Bladder)	Pieris Pharmaceuticals, Inc.	Phase 1	45	Anticalins
		0				NCT03330561	PRS-343 in HER2-Positive Solid Tumors	Recruiting	HER2-positive Solid Tumor (Breast, Gastric, Bladder)	Pieris Pharmaceuticals, Inc.	Phase 1	110	
**BI 836880**	0	1	3	VEGF	Ang2	NCT03972150	A Study to Find the Best Dose of BI 836880 Alone and in Combination With BI 754091 in Japanese Patients With Different Types of Advanced Cancer	Active, not recruiting	Neoplasms	Boehringer Ingelheim	Phase 1	21	Nanobody
		0				NCT02689505	Weekly BI 836880 in Patients With Advanced Solid Tumors	Completed	Neoplasms	Boehringer Ingelheim	Phase 1	24	
		0				NCT02674152	Dose Finding Study of BI 836880 in Patients With Solid Tumors	Completed	Neoplasms	Boehringer Ingelheim	Phase 1	29	
		1				NCT04499352	A Study to Test BI 754091 Alone or in Combination With BI 836880 in People Who Have	Withdrawn	Anal Canal Squamous Cell Carcinoma	Boehringer Ingelheim	Phase 2	0	

							Advanced Anal Cancer						
		1				NCT03468426	A Study to Test Different Doses of BI 836880 Combined With BI 754091 in Patients With Advanced Non-small Cell Lung Cancer Followed by Other Types of Advanced Solid Tumours	Recruiting	Non-squamous, Non-Small-Cell Lung Cancer∣Neoplasms	Boehringer Ingelheim	Phase 1	245	
		1				NCT03697304	Platform Trial Evaluating Safety and Efficacy of BI 754091 Anti-PD-1 Based Combination Therapies in PD-(L)1 naive and PD- (L)1 Pretreated Patient Populations With Advanced/Metastatic Solid Tumours.	Recruiting	Neoplasm Metastasis	Boehringer Ingelheim	Phase 2	260	
**BI 905677**	0	0	2	LRP5/6	LRP5/6	NCT03604445	This Study Aims to Find and Test a Safe Dose of BI 905677 in Patients With Different Types of Cancer (Solid Tumours)	Recruiting	Solid Tumors	Boehringer Ingelheim	Phase 1	78	Nanobody
**MP0274**	0	0	2	HER2	HER2	NCT03084926	First-in-human Study to Investigate Safety, Blood Levels and Activity of MP0274 in Cancer Patients With HER2-positive Solid Tumors	Active, not recruiting	HER2-positive Solid Tumors	Molecular Partners AG	Phase 1	46	DARPin

Table S1. Clinical Trials investigating bsAbs in Solid Malignancies. Table showing all the bsAbs in clinical trials as of June 30th 2021, after interrogation of the ClinicalTrials.gov database using the terms “bispecific”, “antibody”, “solid tumors”, “solid malignancies”, “cancer” and variations of these terms, followed by manual verification for as drug product.

TRIAL/NCT	Agent	Full name	# Patients	Efficacy Readout	Clinical responses	Most Common AE	Reference
CHRYSALIS (NCT02609776)	Amivantamab	Study of Amivantamab, a Human Bispecific EGFR and cMet Antibody, in Participants With Advanced Non-Small Cell Lung Cancer	81 patients	ORR 40% (95% CI, 29-51)<br>DOR 11.1 months (95% CI, 6.9-NR)<br>median PFS 8.3 months (95% CI, 6.5-10.9)	3 CR, 29 PR, 39 SD, 2 NE	Rash (98%)<br>IRR (66%)<br>Paronychia (45%)	46
CHRYSALIS-2 (NCT04077463)	Amivantamab	A Study of Lazertinib as Monotherapy or in Combination With Amivantamab in Participants With Advanced Non-small Cell Lung Cancer	45 patients	ORR 36% (95% CI, 22-51)<br>DOR 9.6 months (95% CI, 5.3-NR)<br>median PFS 4.9 months (95% CI, 3.7–9.5)	1 CR, 15 PR	IRR (78%)<br>Rash (78%)<br>Paronychia (49%)	47
NCT02892123	Zanidatamab	Trial of ZW25 (Zanidatamab) in Patients With Advanced HER2-expressing Cancers	20 patients	ORR 47% (95% CI, 23-72)<br>DOR 6.6 months (95% CI, 3.2-NE)	NA	Diarrhea (45%)<br>IRR (30%)	51
NCT03619681	KN026	Trial of KN026 in Patients With HER2-positive Advanced Malignant Breast Cancer and Gastric Cancer	62 patients	ORR 32.1% (95% CI, 20.3-46)<br>DCR 76.8% (95% CI 63.6-87)	NA	Fever (23.8%)<br>Diarrhea (19%)	54
NCT03925974	KN026	KN026 in Patients With HER2 Expressing Gastric/Gastroesophageal Junction Cancer	18 patients	ORR 55.6% (10/18)<br>DCR 72.2% (13/18)	NA	Transaminase increase (26%)<br>Rash (19%)	55
NCT03321981	Zenocutuzumab	MCLA-128 With Trastuzumab/Chemotherapy in HER2+ and With Endocrine Therapy in ER+ and Low HER2 Breast Cancer	26 patients	DCR 77% (90% CI, 60-89) cohort 1	1 CR, 4 PR (2 unconfirmed)	Neutropenia (61%)<br>Diarrhea (61%)	59
NCT03321981	Zenocutuzumab	MCLA-128 With Trastuzumab/Chemotherapy in HER2+ and With Endocrine Therapy in ER+ and Low HER2 Breast Cancer	42 patients	DCR 45% (90% CI, 32-59) cohort 2	2 PR (unconfirmed), 19 SD	Fatigue (27%)<br>Diarrhea (25%)	60
NCT02912949	Zenocutuzumab	A Study of Zenocutuzumab (MCLA-128) in Patients With Solid Tumors Harboring an NRG1 Fusion	51 patients	ORR 27% (90% CI, 15-43)	NA	NA	62
NCT03330561	Cinrebafusp alfa	PRS-343 in HER2-Positive Solid Tumors	33 patients	ORR 12% (40% at/above doses of 8mg/kg)<br>DCR 52% (70% at/above doses of 8mg/kg)	3% CR, 9% PR, 40% SD<br>10% CR, 30% PR at/above doses of 8mg/kg	IRR (25%)<br>Nausea (7%)	65
NCT00836654	Catumaxomab	Study in EpCAM Positive Patients With Symptomatic Malignant Ascites Using Removab Versus an Untreated Control Group	258 patients	Punture-free survival 46 vs 11 days (p<.0001)<br>Time-to-next paracentesis 77 vs 11 days (p<.0001)	NA	Fever (60.5%)<br>Abdominal pain (43%)	69, 70
NCT03529526	KN046	Study of the Safety, Tolerability, Pharmacokinetics, Immunogenicity and Antitumor Activity of KN046 in Subjects With Advanced Solid Tumors	25 patients	ORR 12% (3/25)<br>DCR 52%<br>median PFS 2.69 months (95% CI, 1.3-5.5)	NA	Pruritus (27.6%)<br>Rash (27.6%)<br>Fatigue (20.7%)	72
NCT03872791	KN046	A Study of KN046 in Subjects With Locally Advanced or	27 patients	median PFS 7.3 months (4.04-NE)	NA	Transaminase increase (48%)	73

		Metastatic Triple-negative Breast Cancer				Fever (33%)	
NCT03852251	Cadonilimab	A Study of AK104, a PD-1/CTLA-4 Bispecific Antibody, for Advanced Solid Tumors or With mXELOX as First-line Therapy for Advanced Gastric or GEJ Adenocarcinoma	34 patients	ORR 66.7% (95% CI, 45-84)<br>DCR 95.8% (95% CI, 79-100)	2 CR, 14 PR	Neutropenia (26.5%)<br>IRR (17.6%)	75
NCT04444167	Cadonilimab	A Study of Anti-PD-1/CTLA-4 Bispecific AK104 Plus Lenvatinib in First-line Advanced Hepatocellular Carcinoma	18 patients	ORR 44% (8/18)<br>DCR 78% (14/18)	NA	Transaminase increase (36.7%)<br>Thrombocytopenia (33%)	76

Table S2. Early Clinical Results of bsAbs in Solid Cancer. Table showing patient characteristics, objective clinical responses and safety parameters of the most representative clinical trials investigating bsAb agents in solid malignancies. Abbreviations: Not Available, NA; Not Estimable, NE; Not Reached, NR; Overall Response Rate, ORR; Disease Control Rate, DCR; Duration of Response, DOR; Adverse Event, AE; Infusion Related Reaction, IRR; Complete Response, CR; Partial Respose, PR; Stable Disease, SD.
